# Disorders of gut microbiota and fecal–serum metabolic patterns are associated with pulmonary tuberculosis and pulmonary tuberculosis comorbid type 2 diabetes mellitus

**DOI:** 10.1128/spectrum.01772-24

**Published:** 2025-03-14

**Authors:** Yunguang Wang, Xinxin He, Yixuan Gao, Mengjiao Xue, Hua Zhang, Lifang Sun, Qiang He, Juan Jin

**Affiliations:** 1 Department of Nephrology, the First Affiliated Hospital of Zhejiang Chinese Medical University (Zhejiang Provincial Hospital of Chinese Medicine), Hangzhou, Zhejiang, China; 2 Zhejiang Key Laboratory of Research and Translation for Kidney Deficiency-Stasis-Turbidity Disease, Hangzhou, Zhejiang, China; 3 Department of Tuberculosis, Zhejiang Hospital of Integrated Traditional Chinese and Western Medicine, Hangzhou, Zhejiang, China; Texas A&M University, College Station, Texas, USA

**Keywords:** pulmonary tuberculosis, diabetes mellitus, gut microbiota, metabolic pathways, fecal metabolites

## Abstract

Pulmonary tuberculosis (PTB) and diabetes mellitus (DM) are prevalent chronic diseases with substantial implications for human health. DM patients are more susceptible to PTB, which exacerbates diabetes-related complications. However, the complex molecular mechanisms underlying the enhanced susceptibility of DM patients to PTB infection remain poorly understood. In this study, α- and β-diversity of gut microbiota was significantly reduced in PTB patients and PTB–DM patients. The abundances of families *Lachnospiraceae* and *Ruminococcaceae* in the the *Firmicutes* phylum were reduced in PTB patients and further diminished in PTB–DM patients. On the other hand, untargeted metabolomics in frozen serum and stool samples indicated that phenylalanine, tyrosine, and tryptophan biosynthesis, metabolites of arginine, proline, tryptophan, and histidine were consistently altered in PTB patients and PTB–DM patients, with significant upregulation of most metabolites. Amino acids like serine, proline, and histidine were both remarkably elevated in PTB and PTB–DM patients. The correlation network analysis reveals the relationships between the shared microbial biomarkers and the shared metabolic pathways. This research contributes to the exploration of pivotal diagnostic biomarkers for both patients with PTB and PTB accompanied by diabetes. Specifically, shared reductions were identified in the genera *g-Roseburia*, *g-Ruminococcaceae_UCG.013*, *g-Ruminococcaceae_NK4A214*, *g-Lachnospiraceae*_*unclassified*, and *g-Firmicutes*_*unclassified* in addition to notable regulation of amino acids, like glycine, serine, and histidine in patients with PTB and PTB–DM. Our study expands the comprehension of the intricate connections linking gut microbiota, fecal metabolites, and serum metabolites in PTB and PTB–DM patients.

**IMPORTANCE:**

This study expands the understanding of the complex links between gut microbiota, fecal metabolites, and serum metabolites in patients with PTB and PTB-DM through multi-omics techniques. It is helpful for us to understand the complex molecular mechanism of increased susceptibility to PTB infection in diabetic patients.

## INTRODUCTION

Tuberculosis (TB) is a chronic infectious disease caused by *Mycobacterium tuberculosis* infection (1). In 2021, an estimated 6.4 million new tuberculosis (TB) cases were identified, resulting in around 1.4 million deaths associated with TB (2). Pulmonary tuberculosis (PTB) is a severe infection initiated by the bacterium *Mycobacterium tuberculosis* (MTB), primarily affecting the lungs but with potential dissemination to other organs (3). Certain conditions that compromise the immune system can heighten the vulnerability to tuberculosis, including diabetes mellitus (4). Studies have demonstrated that individuals with type 2 diabetes possess a 3.59-fold elevated susceptibility to the onset of active PTB in comparison to non-diabetic counterparts (5). Moreover, individuals diagnosed with diabetes mellitus-associated pulmonary tuberculosis (PTB–DM) showcase intensified tuberculosis characteristic symptoms, including heightened severity of coughing, night sweats, and hemoptysis, as compared to those affected by PTB alone (6). In recent years, most research has concentrated on the relationship between glycemic control in PTB–DM patients and treatment outcomes in anti-tuberculosis therapy, yet timely prediction of treatment failure and more in-depth risk analysis remain lacking. Consequently, the dual burden of early prevention and treatment for PTB and DM is garnering increasing attention.

Research has revealed that PTB leads to alterations in the gut microbiota, and a disrupted gut microbiota also increases host susceptibility to PTB (7). A substantial body of evidence obtained from both animal and human studies indicates that infection with MTB can lead to dysbiosis of the gut microbiota, characterized by alterations in the abundance of specific taxa, particularly bacteria that produce short-chain fatty acids (SCFAs), such as *Ruminococcus* and *Bifidobacterium* (8–10). Indole propionic acid produced by gut bacteria has been demonstrated to potentially restrict MTB both *in vitro* and *in vivo* (11). What has been proven is that the gut microbiota plays a role in the development of type 2 diabetes (T2D) (12, 13). Reports suggest that diabetic patients often exhibit moderate gut dysbiosis characterized by decreased levels of SCFA-producing bacteria and increased levels of potentially pathogenic bacteria (14). The mechanisms by which the microbiota influences T2D are diverse; it modulates inflammation, interacts with dietary components, and affects intestinal permeability, glucose and lipid metabolism, insulin sensitivity, and overall energy homeostasis in mammalian hosts (15). A study has indicated that the composition of the gut microbiome might be associated with the host’s immune status related to tuberculosis and that it can regulate Th1/Th17 cell-mediated immune responses, potentially linked to the susceptibility of tuberculosis in patients with diabetes (16). However, the fundamental reasons and mechanisms through which the gut microbiota is involved in the increased susceptibility of diabetic patients to pulmonary tuberculosis (PTB) remain incompletely elucidated. The current diagnosis of PTB–DM and PTB relies on traditional clinical indicators, which fail to predict the risk and contributing factors of PTB in the early stages of the disease (17). Therefore, there is an urgent need to identify more effective biomarkers.

The fusion of metabolomics with gut microbiota analysis has surfaced as a potent means to comprehend disease advancement, unveil governing mechanisms and pivotal elements, establish connections between gut microbiota and metabolites, and unveil potential complex molecular networks. In this prospective observational study, a combination of 16S rDNA (rRNA gene) sequencing, untargeted fecal metabolomics, and serum metabolomics was applied to compare and integrate differences and commonalities in gut microbiota and metabolomic profiles among patients with PTB, PTB–DM, and healthy individuals. This study aims to explore critical diagnostic biomarkers for PTB or PTB coexisting with diabetes, offering a comprehensive insight into the involved molecular responses.

## RESULTS

### The gut microbiota compositions are severely disrupted in PTB–DM patients, and, to a lesser extent, in PTB patients

**Fig 1:**
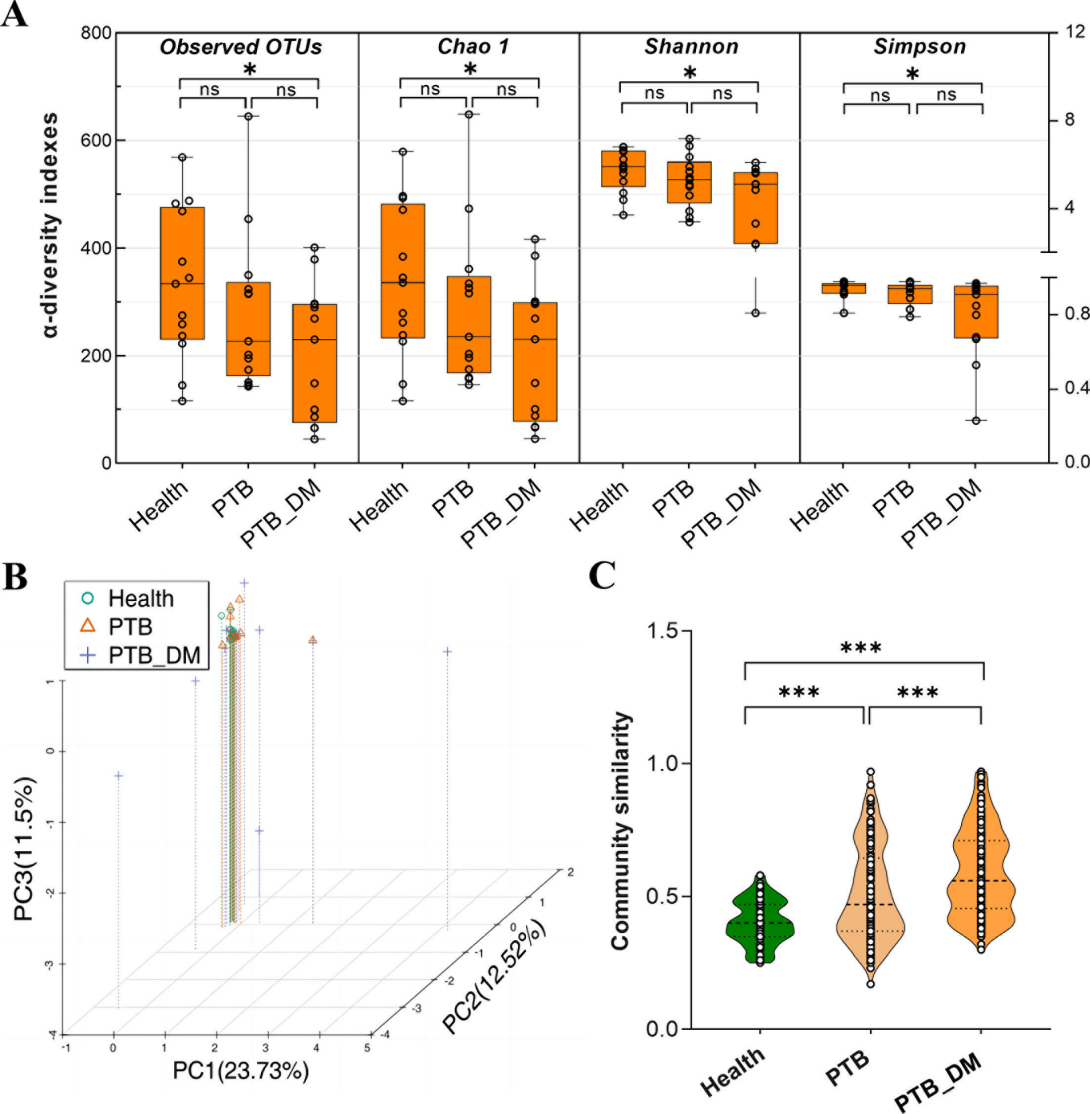
The gut microbiota compositions are severely disrupted in PTB patients and PTB–DM patients. (**A**) α-Diversity indexes, such as observed OTUs, Chao1 diversity index, Shannon and Simpson’s diversity index of intestinal bacteria, were examined by 16S rDNA high-throughput sequencing in each group. (**B**) PCoA analysis based on the relative abundance of amplicon sequence variants (100% similarity), each symbol represents a sample. (**C**) Analysis of bacterial community similarity among the three groups. Health: healthy people, PTB: pulmonary tuberculosis, PTB–DM: pulmonary tuberculosis patients with diabetes mellitus. *P < 0.05, **P < 0.01, ***P < 0.001.

A total of 13 healthy volunteers (Health), 13 pulmonary tuberculosis (PTB) patients, and 13 patients with pulmonary tuberculosis and diabetes mellitus (PTB–DM) were enlisted for the study, and pertinent clinical data were subjected to statistical scrutiny (Table S1) (18). Comparative analysis revealed markedly augmented rates of positive diagnostic indicators in PTB and PTB–DM patients compared with the healthy group (Table S1) (18). In the PTB–DM subset, a noticeable elevation was observed in fever duration exceeding 2 weeks, hemoglobin A1c, and heightened blood glucose levels. Furthermore, PTB–DM patients displayed amplified positive outcomes in GeneXpert MTB/RIF testing, tuberculosis (TB) imaging features, sputum culture, extended fever duration beyond 2 weeks, hemoglobin A1c levels, hemoglobin blood glucose levels, and diabetes tests when contrasted with PTB patients. Characteristic CT images demonstrated centrilobular nodules in the left lungs of PTB patients and nodules with cavitation in both lungs of PTB–DM patients (Fig. S1). These findings inferred that suboptimal diabetes management could potentially exacerbate PTB symptoms and the progression of the disease. To delve into the composition of the gut microbiota within the Health, PTB, and PTB–DM groups, we conducted 16S rDNA gene sequencing. In terms of the richness and diversity of the gut microbiota community, a discernible reduction in the overall α-diversity indices of the PTB group was observed compared with the healthy cohort, whereas the PTB–DM group displayed further diminished α-diversity indices, reaching statistical significance (*P* < 0.05). In contrast, no significant alterations in gut microbiota α-diversity were observed between PTB–DM and PTB patients, indicating that diabetes exacerbates the α-diversity disruption in gut microbiota prompted by PTB (Fig. 1A). Weighted UniFrac phylogenetic distances based on all ASVs were used to measure β-diversity of bacterial communities, and principal component analysis (PCA) illuminated that the discrepancies in community compositions among the three groups primarily emanated from shifts in microbiota compositions among PTB–DM patients (Fig. 1B). Subsequently, community similarity analysis predicated on weighted_unifrac distance underscored marked dissimilarities in gut microbiota compositions between each trio of groups (Fig. 1C). Thus, the compositions of gut microbiota in PTB and PTB–DM patients were significantly different from healthy people, and PTB–DM exacerbated gut microbiota dysbiosis in PTB.

### The composition of microbiota in PTB–DM is distinct from PTB

**Fig 2:**
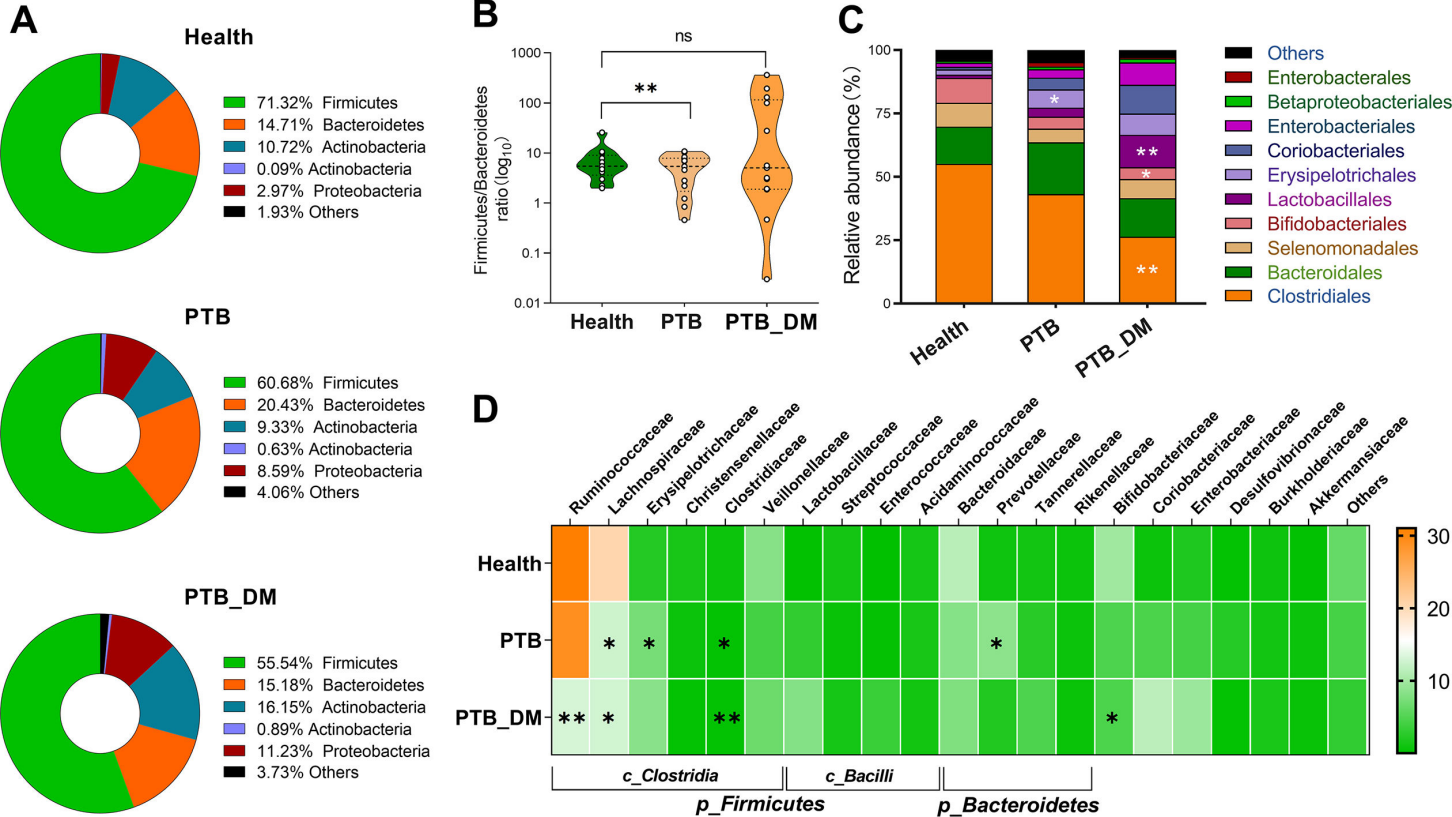
The composition of microbiota in PTB–DM is distinct from PTB. (**A and B**) Relative abundance of the most predominant phyla in various sample groups was visualized with donut chart (**A**) and column chart (**B**). (**C**) The relative abundance heatmap of gut microbiota at the order level was shown. (**D**) The relative abundance heatmap of gut microbiota at the family level was shown. Health: healthy people, PTB: pulmonary tuberculosis, PTB–DM: pulmonary tuberculosis patients with diabetes mellitus. * indicated the significance for PTB or PTB–DM versus Health and # means the significance for PTB–DM versus PTB. *P < 0.05, **P < 0.01, ***P < 0.001.

The human gut microbiota is dominated by *Firmicutes*, *Bacteroidetes*, *Actinobacteria*, *Proteobacteria*, and *Verrucomicrobia*, and their abundance variations are closely related to the host’s physiological status. In this study, the relative abundances of *Firmicutes* and *Bacteroidet*es were 71.32% and 14.71% in the Health group, 60.68% and 20.43% in PTB patients, and 55.54% and 15.18% in PTB–DM patients, respectively (Fig. 2A). The abundance of *Actinobacteria* was significantly elevated in the PTB–DM group compared with the Health and PTB groups (Fig. 2A). Notably, the *Firmicutes/Bacteroidetes* ratio in the PTB–DM group was significantly elevated compared with that in the Health and PTB groups (Fig. 2B). It has been widely reported that the *Firmicutes/Bacteroidetes* ratio is significantly increased in gut microbiota of obese and diabetic populations, along with alterations in host metabolic functions (19). At the order level, the growth of *Clostridiales* and *Bifidobacteriales* was reduced in PTB patients and further reduced in PTB–DM patients, with significant differences. Additionally, patients in the PTB group exhibited a notable elevation in *Lactobacillales* and *Erysipelotrichales*, showing significant differences between the PTB–DM group. These results suggested an enhanced inhibitory effect of DM + PTB on beneficial symbiotic orders, such as *Clostridiales* and *Bifidobacteriales*, while promoting the growth of *Lactobacillales* and *Erysipelotrichales* enriched in PTB (Fig. 2C). At the family level, the results indicated a specific suppression of *Ruminococcaceae* in PTB–DM patients when compared with either healthy individuals or PTB patients. In addition, PTB and PTB–DM patients consistently inhibited the growth of *Clostridiaceae*. Besides, PTB uniquely and significantly decreased the abundance of *Prevotellaceae*. In comparison, PTB + DM uniquely and significantly altered the abundance of *Desulfovibrionaceae* and *Fusobacteriaceae* (Fig. 2D). Taken together, the composition of gut microbiota in PTB–DM patients is distinct from that in PTB patients.

### Differential microbial biomarkers were identified with LEfSe

**Fig 3:**
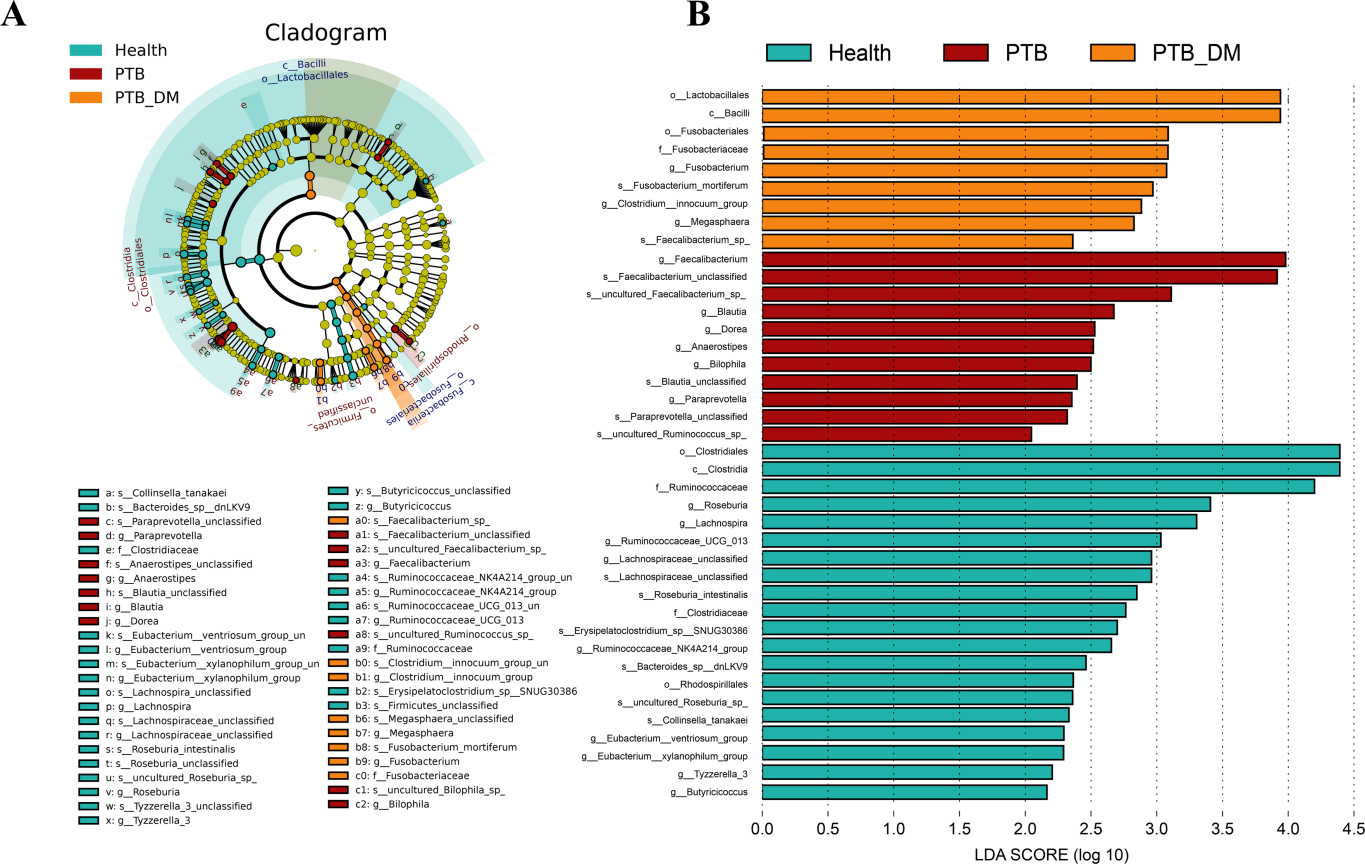
Identification of differential microbial biomarkers. (**A**) Cladogram visualized the most differentially abundant taxa identified by LEfSe among the three groups. Blue indicates clades enriched in the Health group, red indicates clades enriched in the PTB group, and orange indicates clades enriched in the PTB-DM group. (**B**) Comparisons of microbiota bacteria among the three groups. The histogram showed the LDA score computed for genera differentially abundant between groups and identified using LEfSe. Health: healthy people, PTB: pulmonary tuberculosis, PTB-DM: pulmonary tuberculosis patients with diabetes mellitus.

Metastat-based and linear discriminant analysis (LDA) effect size (LEfSe) analyses were performed to identify different distributions of the fecal microbiota. According to the LDA score, as shown in Fig. 3A and B, 20 taxa, including Eubacterium, Ruminococcaceae, and Lachnospiraceae, were abundant in the Health group. In comparison, the optimal-enriched taxa in the stool microbiome of the PTB group were *g_Faecalibacterium*, *g_Blautia*, *g_Dorea*, *g_Bacteroides*, *g_Paraprevotella*, *g_Bilophila*, whereas *Lactobacillales*, *Bacilli*, *Fusobacterium*, and *Megasphaera* and were more abundant in the PTM-DMs (Fig. 3A and B). Furthermore, we also evaluated the capacity of genus features to predict the host status by conducting random forest-based machine learning (Fig. S2). The mean decrease in accuracy (Fig. S2A) and Gini index (Fig. S2B) reflected the importance of each genus, indicating the reductions in prediction accuracy and the percentage of predictions when that genus was removed. As expected, the important genera with higher accuracy or Gini values corresponded to the differential genera identified by LEfSe analysis, including *g_Clostridium_innocuum_group*, *g_Tyzzerella_3*, *g_Bilophila*, *g_Lachnospiraceae_unclassified*, *g_Blautia*, and *g_Faecalibacterium*. However, the predictive performance of the top 20 genera demonstrated unsatisfactory accuracy in discriminating host status (Fig. S2C). In particular, four PTB samples were incorrectly predicted as either healthy or PTB–DM samples. These LEfSe-detected taxonomic biomarkers were further used for the network analysis to dissect the associations between microbiota and fecal and serum metabolomes.

### Fecal metabolome is altered in PTB and PTB–DM patients

**Fig 4:**
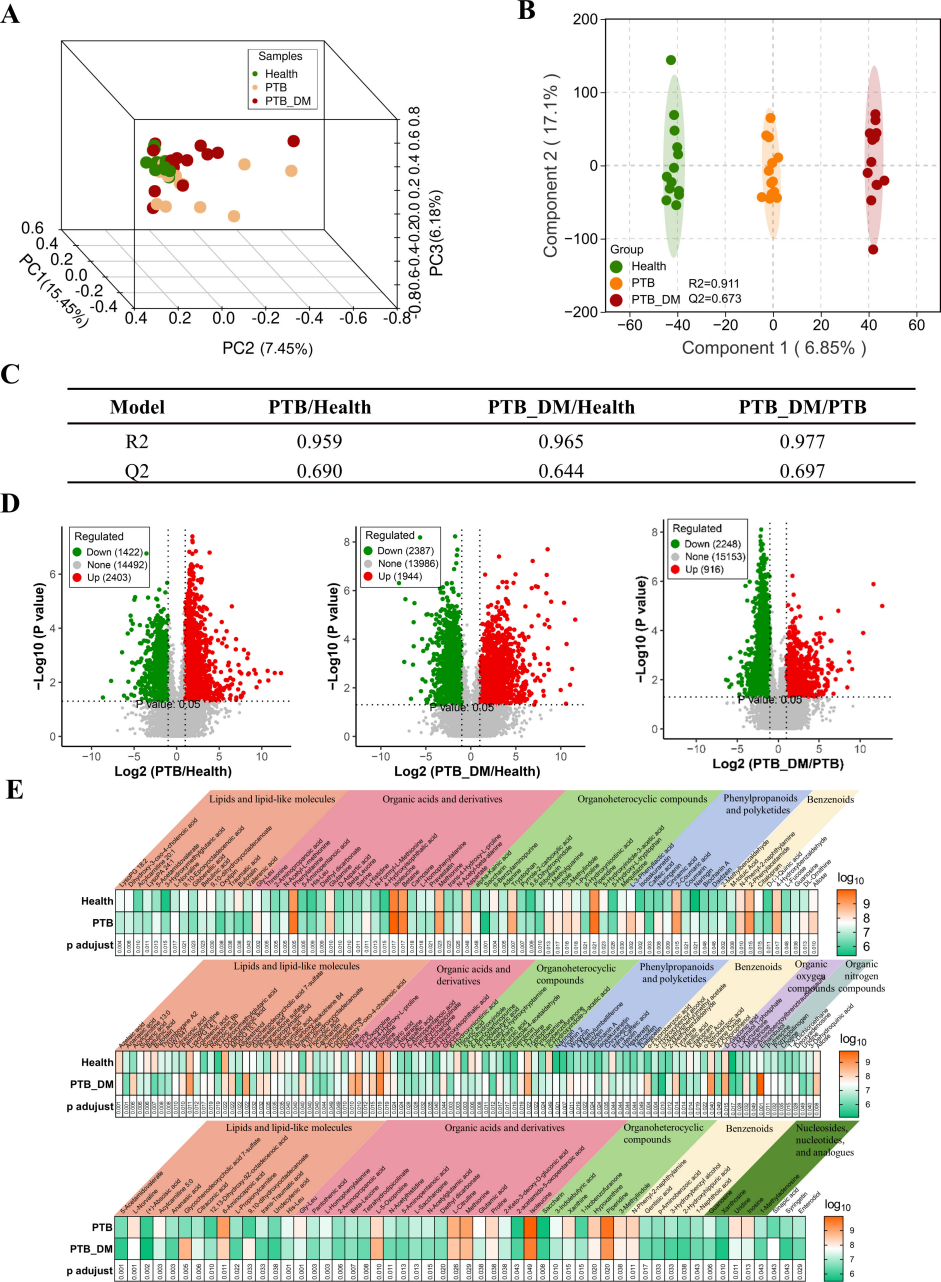
The fecal metabolome was significantly altered in PTB and PTB-DM patients. (**A**) Principal component analysis (PCA) was used to measure the shift in fecal metabolomics among three groups. (**B**) Partial least squares discrimination analysis (PLS-DA) plots derived from UPLC-QTOF-MS metabolite profiles among the three groups. (**C**) R2Y and Q2 values of OPLS-DA model between each two groups.(**D**) Volcano plot of differentially expressed fecal metabolites between the Health group and PTB group (left), Health group and PTB–DM group (middle), and PTB group and PTB–DM group (right). (**E**) The relative abundance of fecal metabolites was depicted by the heatmap between the Health group and PTB group (up), Health group and PTB–DM group (middle), and PTB group and PTB–DM group (bottom). Health: healthy people, PTB: pulmonary tuberculosis, PTB–DM: pulmonary tuberculosis patients with diabetes mellitus.

The principal component analysis score plots in the positive (POS) and negative (NEG) modes clearly distinguished between the Health, PTB, and PTB–DM groups (Fig. 4A), indicating the significant differences among these groups. Next, the R2Y and Q2 for the orthogonal partial least squares discriminant analysis (OPLS-DA) model were 0.911 and 0.673, indicating a reliable and stable model (Fig. 4B). Moreover, the R2Y and Q2 values of the OPLS-DA model between each two groups were both >0.5. Furthermore, differentially expressed metabolites were screened using variable importance in projection (VIP) >1 and *P* < 0.05 as the thresholds (Table S2), and it could be observed from the volcano plot that PTB caused the significant upregulation of 2,403 metabolites and significant downregulation of 1,422 metabolites compared with the Health group. The 2,387 metabolites were significantly upregulated, and 1,944 metabolites were significantly downregulated in PTB–DM patients. Interestingly, PTB–DM led to significant reductions of more metabolites than elevated metabolites at 2,248 and 916, respectively (Fig. 4C). We further annotated the chemical identification of these differential metabolites, which mostly concentrated in lipids and lipid-like molecules, organic acids and derivatives, and organoheterocyclic compounds (Fig. 4D). For the PTB group versus the Health group, 77 differentially expressed metabolites were identified, of which 56 metabolites were upregulated concentrated in organic acids and derivatives, and 21 metabolites were downregulated and concentrated in lipid and lipid-like molecules. For the PTB–DM group versus theHealth group, 100 differentially expressed metabolites were identified, of which 59 metabolites were upregulated and distributed in various classes, and 41 metabolites were downregulated and also concentrated in lipid and lipid-like molecules (Table S2). Furthermore, 54 differentially expressed metabolites were responsible for the distinction between the PTB–DM and PTB groups. Most of the metabolites were downregulated, and only nine metabolites were elevated. Collectively, these results demonstrated that the that fecal metabolome was robustly altered in PTB and PTB–DM patients.

### KEGG metabolic pathway analysis of fecal metabolomics

**Fig 5:**
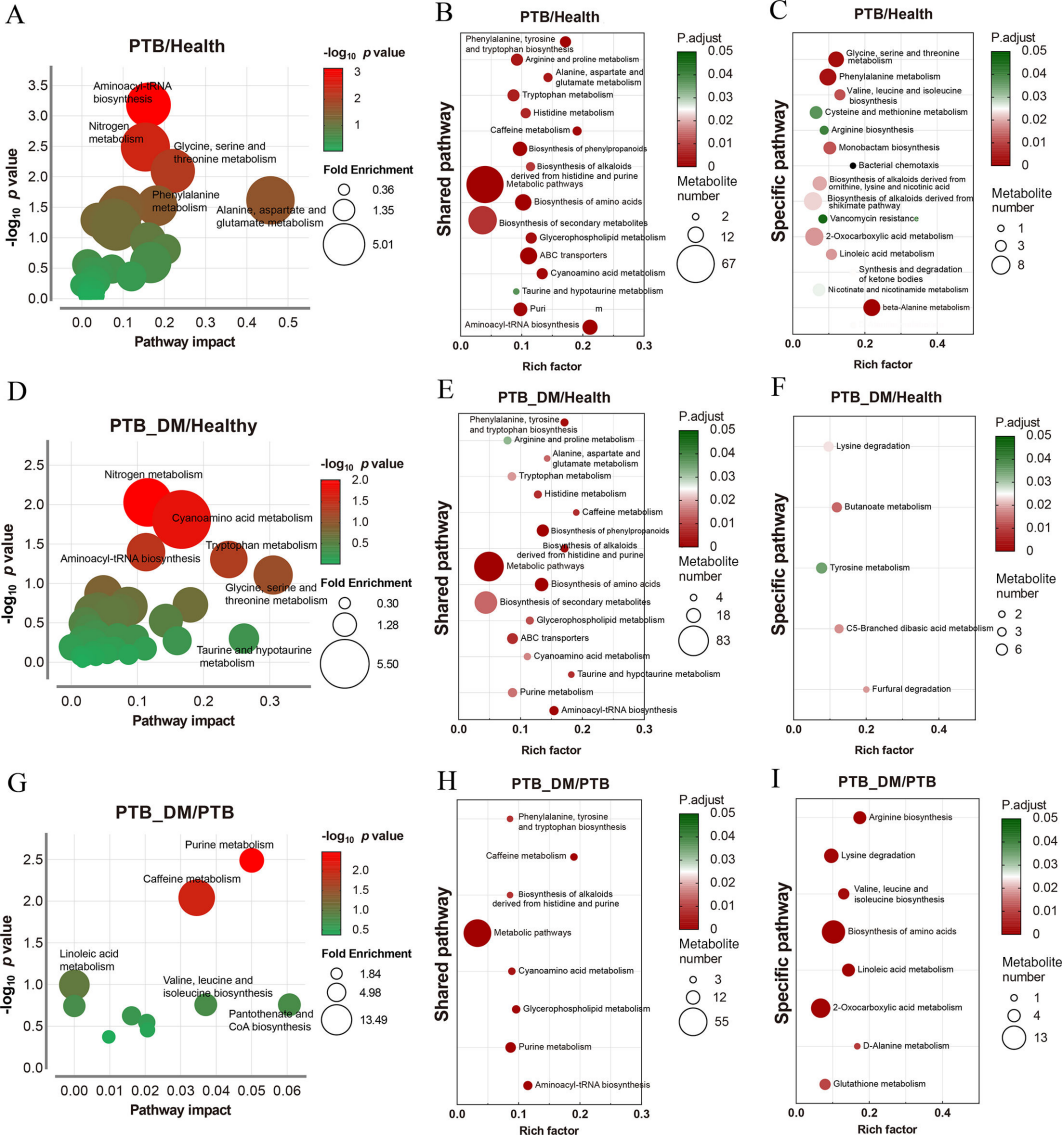
KEGG metabolic pathway analysis of fecal metabolomics. (**A, D, G**) Overview of pathway analysis, indicating significantly enriched metabolic pathways for differential metabolites between the Health group and PTB group (**A**), Health group and PTB-DM group (**D**), and PTB group and PTB-DM group (**G**). (**B, E, H**) The bubble charts depicted the degree of enrichment in shared KEGG metabolic pathways between the Health group and PTB group (**B**), Health group and PTB-DM group (**E**), and PTB group and PTB-DM group (**H**). (**C, F, I**) The bubble charts depicted the degree of enrichment in specific regulated KEGG metabolic pathways between the Health group and PTB group (**C**), Health group and PTB-DM group (**F**), and PTB group and PTB-DM group (**I**). Health: healthy people, PTB: pulmonary tuberculosis, PTB-DM: pulmonary tuberculosis patients with diabetes mellitus. Bubble size represents identified differential metabolites, while the rich factor indicates the ratio of identified differential metabolites to all metabolites within that KEGG pathway.

The results of the pathway study are presented in the form of bubble plots. Regulation of a total of 33 potential pathways, such as aminoacyl-tRNA biosynthesis, phenylalanine metabolism, and glycine, serine, and threonine metabolites were observed between the PTB and Health groups, and 22 pathways, such as cyanoamino acid metabolism and tryptophan metabolism, were enriched between the PTB–DM and Health groups (Fig. 5A and D; Table S3). Besides, the pathways shared between the PTB and PTB–DM groups were phenylalanine, tyrosine and tryptophan biosynthesis, arginine and proline metabolism, alanine, aspartate, and glutamate metabolism, and tryptophan metabolism (Fig. 5B and E; Table S3). Specific pathways in the PTB group/Health group were mainly amino acid metabolism, such as phenylalanine metabolism, valine, leucine, and isoleucine biosynthesis (Fig. 5C; Table S3), while less specific pathways were enriched in the PTB–DM group/Health group, such as lysine degradation, tyrosine metabolism, and butanoate metabolism (Fig. 5F; Table S3). Moreover, a total of 16 pathways were enriched between the PTB–DM and PTB groups, such as purine metabolism and caffeine metabolism (Fig. 5G, Table S3). Besides, shared pathways in the PTB–DM group/PTB group were phenylalanine, tyrosine and tryptophan biosynthesis, caffeine metabolism, and metabolic pathways (Fig. 5H; Table S3). Specific pathways were arginine metabolism, lysine degradation, and biosynthesis of amino acids (Fig. 5I; Table S3). Together, for the fecal metabolome, most of the significantly altered metabolic pathways in the PTB–DM patients were shared with PTB patients; meanwhile, 7 of 16 shared metabolic pathways were significantly altered between PTB and PTB–DM patients.

### Serum metabolomics analysis

**Fig 6:**
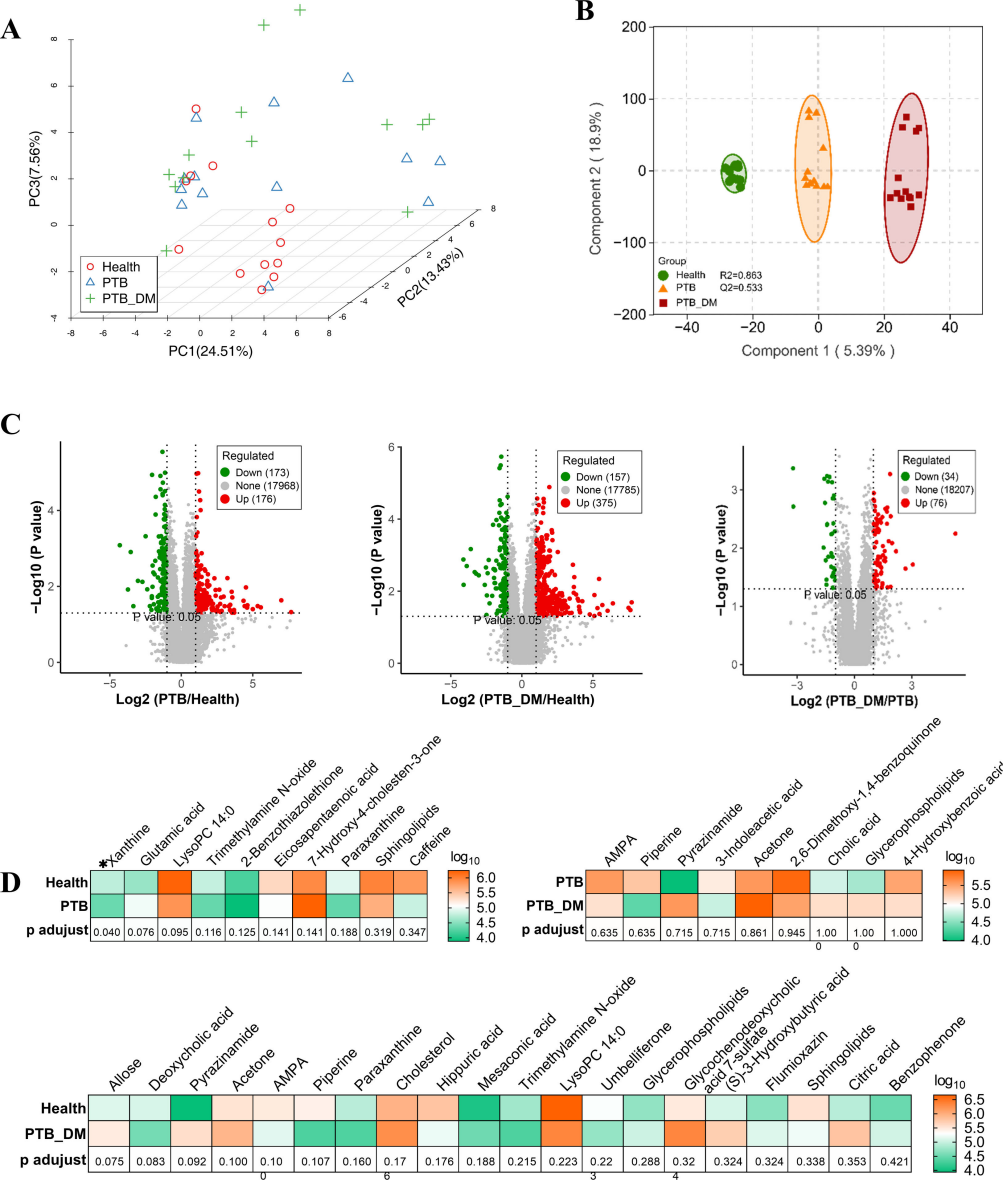
Serum metabolomic analysis. (**A**) Principal component analysis (PCA) was used to measure the shift in serum metabolomics among three groups. (**B**) Partial least squares discrimination analysis (PLS-DA) plots derived from UPLC-QTOF-MS metabolite profiles among three groups. (**C**) Volcano plot of differentially expressed serum metabolites between Health and PTB groups (left), Health group and PTB–DM group (middle) and PTB group and PTB–DM group (right). (**D**) The relative intensity of differentiated serum metabolites was depicted by the heatmap between the Health group and PTB group (up), Health group and PTB–DM group (middle), and PTB group and PTB–DM group (bottom). Health: healthy people, PTB: pulmonary tuberculosis, PTB–DM: pulmonary tuberculosis patients with diabetes mellitus.

To observe the changes in the serum metabolic profiles of PTB–DM patients and PTB patients, we conducted serum metabolomics experiments. PCA was utilized to show the difference of serum metabolites among the three groups (Fig. 6A). OPLS-DA was performed to visualize clustering patterns of the metabolic profiles and identify differential metabolites between groups (Fig. 6B). OPLS-DA score plots revealed distinct clustering of metabolic profiles within groups with R2Y = 0.863 and Q2 = 0.533. However, there was a subtle difference between the PTB–DM and PTB groups with R2Y = 0.927 and Q2 = −0.007. Volcano plots showed that 173 metabolites were upregulated, and 176 metabolites were downregulated in PTB patients compared with the Health group, and 157 upregulated metabolites and 375 downregulated metabolites were found in PTB–DM patients compared with the Health group (Fig. 6C; Table S4). However, for the PTB–DM group versus PTB group, fewer metabolites were detected, with 34 metabolites upregulated and 76 downregulated (Table S4). Differential metabolites between groups were further identified using VIP >1.0 and *P*  <  0.05 as standards. Heatmaps displayed the level of differential metabolites (Fig. 6D). On the whole, fewer metabolites were identified in serum metabolome compared with fecal metabolome. Ten metabolites, such as xanthine (*P* < 0.05) and glutamic acid were screened between the PTB and Health groups, 20 metabolites, such as allose, deoxycholic acid, and pyrazinamide, for the PTB–DM and Health groups, and nine metabolites for the PTB–DM and PTB groups. On the whole, fewer differential serum metabolites were found compared with the fecal metabolome.

### KEGG metabolic pathway analysis of serum metabolomics

**Fig 7:**
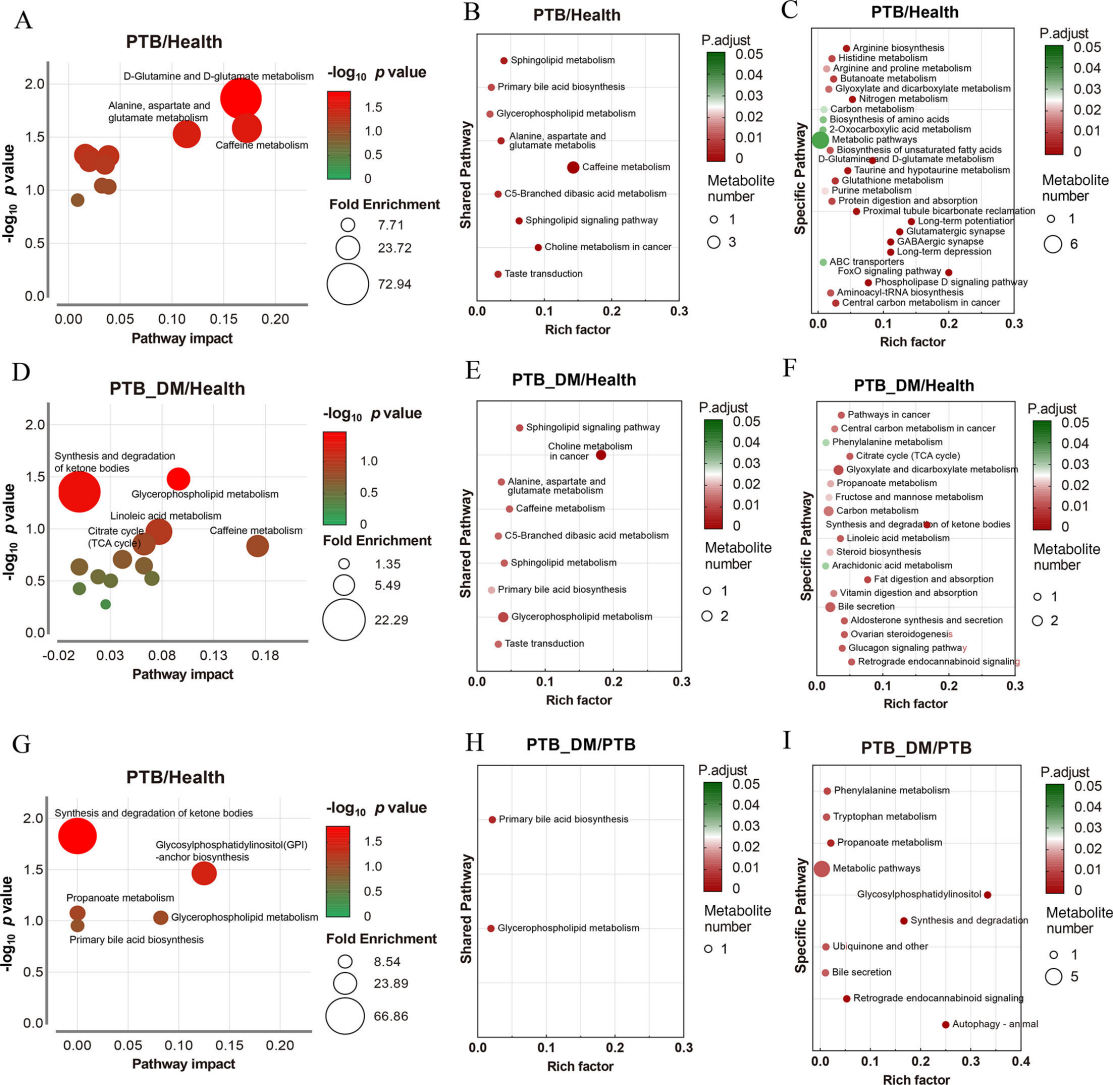
KEGG metabolic pathway analysis of serum metabolomics. (**A, D, G**) Overview of pathway analysis, indicating significantly enriched metabolic pathways for differential metabolites in serum between the Health group and PTB group (**A**), Health group and PTB–DM group (**D**), and PTB group and PTB–DM group (**G**). (**B, E, H**) The bubble charts depicted the degree of enrichment in shared KEGG metabolic pathways between the Health group and PTB group (**B**), Health group and PTB–DM group (**E**), and PTB group and PTB–DM group (**H**). (**C, F, I**) The bubble charts depicted the degree of enrichment in specific regulated KEGG metabolic pathways between the Health group and PTB group (**C**), Health group and PTB–DM group (**F**), and PTB group and PTB–DM group (**I**). Health: healthy people, PTB: pulmonary tuberculosis, PTB–DM: pulmonary tuberculosis patients with diabetes mellitus. Bubble size represents identified differential metabolites, while the rich factor indicates the ratio of identified differential metabolites to all metabolites within that KEGG pathway.

Similarly, bubble plots showed the enriched metabolic pathways. Pathways such as D-glutamine and D-glutamate metabolism, alanine, aspartate and glutamate metabolism, and caffeine metabolism were observed between the PTB and Health groupa, and pathways, such as synthesis and degradation of ketone bodies, were enriched between the PTB–DM and Health groups (Fig. 7A and D; Table S5). Besides, the pathways shared between the PTB/Health and PTB–DM/Health groups were the sphingolipid signaling pathway, choline metabolism in cancer, alanine, aspartate and glutamate metabolism, and caffeine metabolism (Fig. 7B and E; Table S5). The specific pathways in the PTB group/Health group were arginine biosynthesis, histidine metabolism, and arginine and proline metabolism (Fig. 7C; Table S5). The specific pathways in the PTB–DM group/Health group were the pathways in cancer, central carbon metabolism in cancer, and phenylalanine metabolism (Fig. 7F; Table S5). However, fewer pathways were enriched between the PTB–DM and PTB groups, such as synthesis and degradation of ketone bodies and glycosylphosphatidylinositol (GPI)-anchor biosynthesis (Fig. 7G; Table S5). Besides, shared metabolic pathways in the PTB–DM group/PTB group were primary bile acid biosynthesis and glycerophospholipid metabolism (Fig. 7H; Table S5). The specific pathways were phenylalanine metabolism, tryptophan metabolism, and metabolic pathways (Fig. 7I; Table S5). Overall, for the serum metabolome, the majority of the significantly altered metabolic pathways in PTB–DM patients were distinguished from those in PTB patients. Meanwhile, only two of nine shared metabolic pathways were remarkably altered between PTB and PTB–DM patients.

### Deconfounding analysis of omics features and clinical variables and correlation analysis between omics features

**Fig 8:**
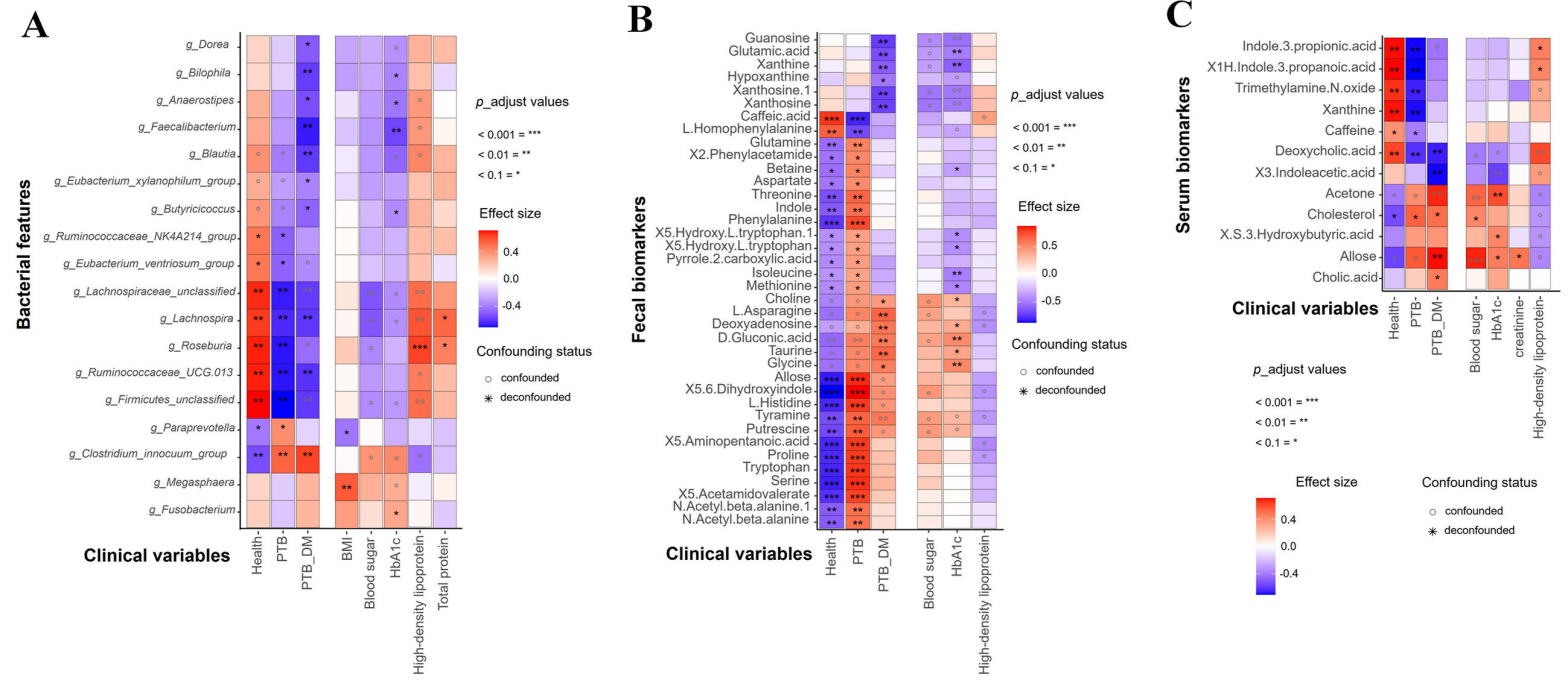
Deconfounding analysis of omics features and clinical variables and correlation analysis between omics features. MetadeconfoundR was used for analysis. The cofounded results are denoted by a circle, while deconfounded results are represented by a star. The heatmap illustrates confounders or non-confounders and their relationships with differential genus (**A**), differential fecal metabolites (**B**), and differential serum metabolites (**C**). Significance assessment was carried out using Mann–Whitney U for two categorical variables and Spearman for continuous variable test, *FDR < 0.1, **FDR < 0.01, ***FDR < 0.001.

The deconfounding analysis results between the differential genus and clinical parameters revealed that the associations were confounded by levels of HbA1c, blood sugar, and high-density lipoprotein. Specifically, both HbA1c and blood sugar exhibited positive correlations with *g_Clostridium_innocuum_group* and *g_Megasphaera* (identified as biomarkers in PTB–DM) and negative correlations with *g_Lachnospira* and *g_Lachnospiraceae_unclassified* (identified biomarkers in the healthy group) (Fig. 8A). Furthermore, health status and high-density lipoprotein demonstrated similar impacts on these differential genera. The findings from the deconfounding analysis between metabolite features and clinical parameters are depicted in Fig. 8B and C. Notably, the PTB–DM status and blood sugar presented a more consistent pattern in altering metabolite features in the fecal metabolome compared with the serum metabolome (Fig. 8B and C). The new Fig. 8 has been presented below.

### Correlation analysis of gut microbes, fecal metabolites, and serum metabolites

**Fig 9:**
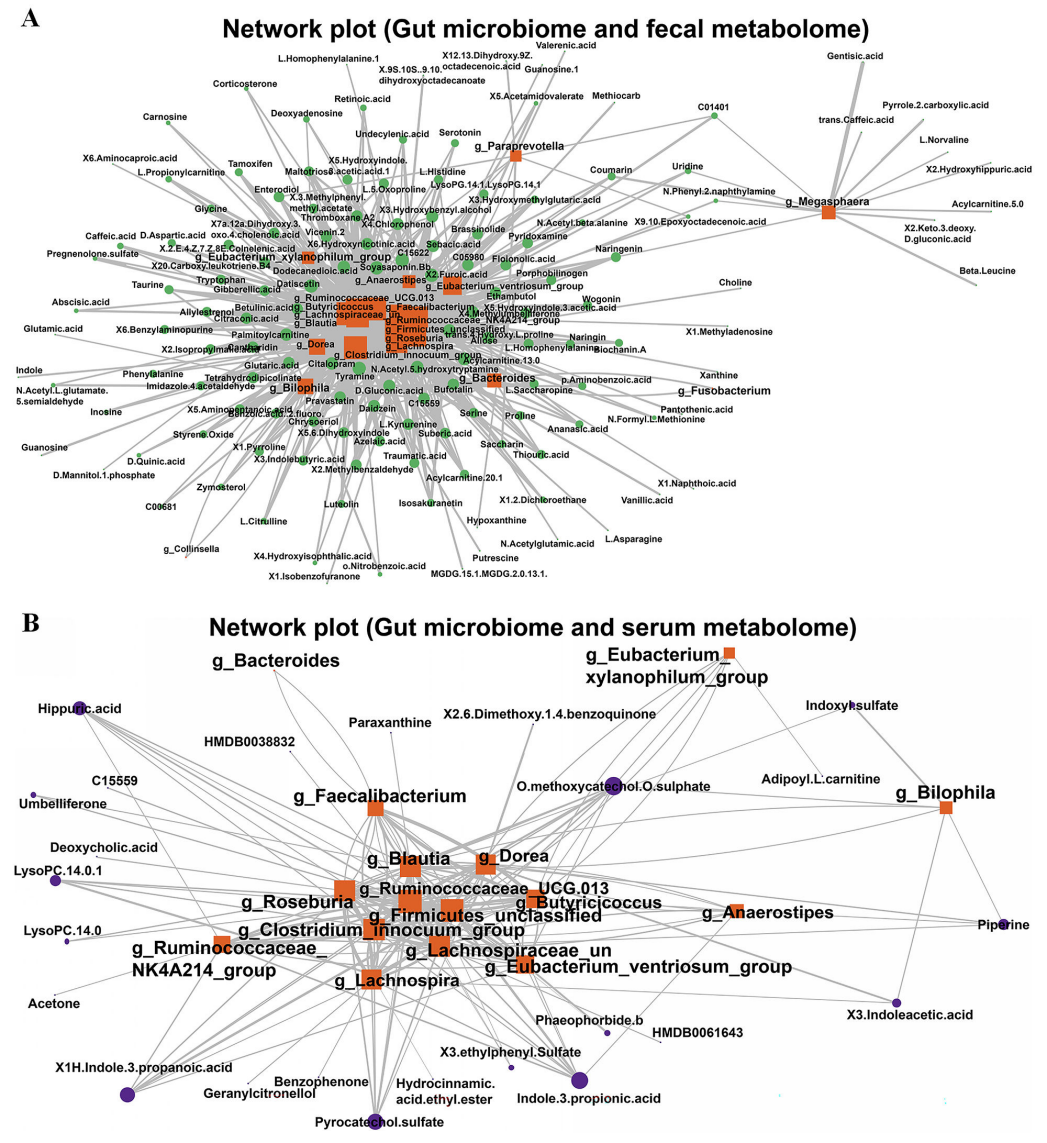
Gut microbiota was correlated with fecal metabolome and serum metabolome, respectively. Correlation network analysis between microbial biomarkers and differential fecal metabolites (**A**) and between microbial biomarkers and differential serum metabolites (**B**).

To further explore the potential relationships between altered gut microbiota and fecal and serum metabolomes, we performed correlation analysis between these identified taxonomically genus biomarkers and differential fecal and serum metabolites. It was found that gut microbiota in the orange labels correlated more tightly with fecal metabolites in green labels (Fig. 9A) than serum metabolites in purple labels (Fig. 9B). The results showed that a total of 17 microbial communities at the genus level showed significant correlations with fecal differential metabolites. For example, *g_Magasphaera* was tightly correlated with gentisic acid. Though less correlation was found between gut microbiota and serum metabolites, there is a significant correlation between 15 genera and specific differential metabolites. For instance, *g_Blautia* correlated tightly with *paraxanthine*, and *g-Ruminococcaceae_NK4A214* had a correlation with acetone. In conclusion, in PTB–DM and PTB patients, there were tight correlations among gut microbiota, fecal metabolites, and serum metabolites.

### Correlations between microbial biomarkers and differentiated fecal metabolites

Next, we performed an analysis on gut microbiota and gut metabolites, and it was found that PTB and PTB–DM both downregulated *g_Roseburia*, *g_Ruminococcaceae_UCG.013*, *g-Ruminococcaceae_NK4A214*, *g-Lachnospiraceae_unclassified*, and *g-Firmicutes*_*unclassified. g_Paraprevotella* was specifically enriched in PTB but without statistical significance. Besides, *g-Faecalibacterium*, *g-Lachnospira*, and *g-Butyricicoccus* were specifically enriched in PTB–DM (Fig. S3). For fecal metabolomics, the pathways shared between the PTB and PTB–DM groups were phenylalanine, tyrosine and tryptophan biosynthesis, arginine and proline metabolism, tryptophan metabolism, histidine metabolism, biosynthesis of amino acids and ABC transporters. Consistent with that, the common differentially expressed metabolites regulated by PTB–DM and PTB were mainly amino acids, such as proline, tryptophan, L-homophenylalanine, serine, L-histine, tryamine, betaine, and specific pathways in the PTB group/Health group were glycine, serine and threonine metabolism, cysteine and methionine metabolism, arginine biosynthesis, and beta-alanine metabolism, mainly containing specific metabolites, such as aspartate, threonine, gluamine, methionine, pyrrole-2-carboxylic and N-acetyl-beta-alanine. On the other hand, specific metabolic pathways were enriched in the PTB–DM group/Health group, such as purine metabolism, lysine degradation, taurine and hypotaurine metabolism and metabolic pathways (Fig. S3). Glycine, xanthine, guanosine, xanthosine, deoxyadenosine, choline, L-asparagine, and taurine were found only regulated in the PTB–DM group (Fig. S3). Therefore, the shared fecal microbial biomarkers and metabolic pathways may be developed as diagnostic biomarkers for predicting the risk of developing PTB in DM patients.

Next, the Dirichlet-Multinomial (v 1.46.0) R package was employed to establish the grouping patterns of the samples at the genus level. We found that a total of 18 core genera that exhibit prevalence at 0.1% relative abundance in 50% of the samples were identified (Fig. S4A). However, our 16S rRNA data sets, whether encompassing all samples or only selected ones, failed to cluster into distinct microbial community types due to the similar and low values of cluster importance among the genus drivers. The main drivers for community type 1 were identified as *g_Faecalibacterium*, *g_Bacteroides*, and *g_Subdoligranulum*, indicating their inadequacy in stratifying PTB–DM from PTB. Interestingly, community type 2 in PTB–DM samples was characterized by the dominance of *g_Erysipelatoclostridium* (Fig. S4B), while only two PTB–DM samples exhibiting high abundance of *g_Erysipelatoclostridium*, alongside the PTB_5 sample (Fig. S4C).

### Correlations between microbial biomarkers and differentiated serum metabolites

Next, we next focused on serum metabolic features regulated in the PTB and PTB–DM groups. The results showed that only three differentiated serum metabolites were shared in the PTB group and PTB–DM group, which were trimethyamine N-oxide, deoxycholic acid, and indole-3-propionic acid. However, no shared metabolic pathway had been identified based on them. The specific serum metabolic pathway for PTB was only one, caffeine metabolism, including two metabolites, xanthine and caffeine. For PTB–DM, primary bile acid biosynthesis, including cholesterol and cholic acid, tryptophan metabolism and synthesis, including indole-3-acetate metabolites, and synthesis and degradation of ketone bodies, including acetone were specific pathways (Fig. S5).

## DISCUSSION

PTB infection remains a major threat to health, and potential biomarkers are advantageous for the diagnosis and treatment of PTB in the clinical setting (20). Some studies have reported patients with both PTB and DM suffer more severe PTB infections (21). In this study, by combining approaches, such as gut microbiota and metabolomics, we revealed the characteristic compositions of gut microbiota, fecal metabolites, and serum metabolite profiles in PTB and PTB–DM patients. Through comprehensive analysis, we found significant differences in gut microbiota and fecal metabolites among healthy individuals, PTB patients, and PTB–DM patients. However, PTB and PTB–DM could not be fully distinguished based on serum metabolites. We also identified common differentially abundant genera, including *g-Roseburia*, *g-Ruminococcaceae_UCG.013*, *g-Ruminococcaceae_NK4A214*, *g-Lachnospiraceae_unclassified*, and *g-Firmicutes_unclassified* in PTB and PTB–DM. *G_Paraprevotella* might contribute to shared differential fecal metabolites and pathways, such as proline, tryptophan, L-homophenylalanine, serine, L-histine, tryamine, and betaine. Amino acid metabolism could be a significant metabolic process in both PTB and PTB–DM.

The gut microbiota undergoes significant dysbiosis due to PTB and anti-tuberculosis treatment (22). In comparison to healthy controls, PTB infection by *M. tuberculosis* leads to a slight decrease in gut microbiota α-diversity, primarily attributed to alterations in the relative abundance of members of the *Bacteroides* genus (7). Consistent with these findings, our results reveal that tuberculosis infection induces significant changes in the diversity and composition of the gut microbiota. Evidence suggests a link between diabetes-associated gut dysbiosis and modulated host immune response to *M. tuberculosis* infection (23). The α-diversity of gut microbial communities in the PTB–DM group was further reduced. *Actinobacteria* and *Firmicutes* are the major phyla in the gut microbiota. Imbalances in the *Actinobacteria*-to-*Firmicutes* ratio indicate disrupted gut homeostasis, pathogen invasion, or unhealthy status (24). Several independent research groups have observed a significant reduction of the Firmicutes phylum in tuberculosis patients (10), which might also be triggered by the dysregulated immune system caused by MTB infection. Our results also indicated a significant downregulation of the *Firmicutes* phylum in PTB. The ratios of *Bacteroidetes* to *Firmicutes* correlated positively and significantly with plasma glucose values (25). The *Firmicutes* phylum was further diminished in PTB–DM patients, and the *Bacteroidetes*-to-*Firmicutes* ratio was further elevated compared with PTB patients. In PTB patients, many observations support the reduction of families *Lachnospiraceae*, *Ruminococcaceae*, and *Clostridiaceae* within *Clostridiales* in *Firmicutes* and *Prevotella* in *Bacteroidetes* (26, 27). Consistent with the results mentioned above, at the family level, *Lachnospiraceae*, *Ruminococcaceae,* and *Clostridiaceae* were both downregulated in PTB and PTB–DM patients compared with healthy people. *Lachnospiraceae* and *Ruminococcaceae* are obligate anaerobic and butyrate-producing bacteria (28). These evidences indicated that butyrate might be regulated in PTB patients and PTB–DM patients. At the genus level, more genera were abundant in healthy people. Genera, such as *Lactobacillales*, *Bacilli,* and *Fusobacteriales*, were enriched in PTB patients, while genera, such as *Faecalibacterium*, *Dorea*, and *Paraprevotella*, were abundant in PTB–DM patients. The changes in genera remained various and needed more investigations. Interestingly, it was found that common genera shared by PTB patients and PTB–DM patients were mainly *Firmicutes*, including *g-Ruminococcaceae_UCG.013*, *g-Ruminococcaceae_NK4A214*, *g-Lachnospiraceae_unclassified* and *g-Firmicutes*_*unclassified*, and these might be attributed to M.tb infection. *g-Faecalibacterium* was reported to be negatively correlated with T2D (13). *g-Faecalibacterium*, *g-Lachnospiraceae*, and *g-Butyricicoccus*, as specific regulated genera in PTB–DM patients, might correlate with diabetes. In addition to that, different community types of the airway microbiome identified by Dirichlet Multinomial (DMM) model can distinguish between active *Mycobacterium tuberculosis* (M.tb) patients and individuals without M.tb infection or with latent M.tb infection. Specifically, we selected a total of 18 core genera prevalent at 0.1% relative abundance in 50% of the samples. Unfortunately, the DMM, whether applied to all samples or specific subsets, was unable to cluster into distinct microbial community types due to the similar and low cluster importance values among the genus drivers. Notably, community type 2 in PTB–DM samples was predominantly composed of *g_Erysipelatoclostridium*, while with limited PTB–DM samples showing high abundance of this genus, as well as a PTB sample.

The gut microbiota plays a crucial role in human health and disease, exerting both beneficial and detrimental effects on essential physiological processes through its metabolites, such as SCFAs, trimethylamine-N-oxide (TMAO), bile acids (BAs), polyphenols, and others (29–31). It has been reported that amino acids are tightly correlated with tuberculosis infection, such as leucine, isoleucine, valine, phenylalanine, aspartate, and glutamate, which were found to be at higher levels in TB patients (32). In our results, many organic acids and derivatives were both upregulated, such as serine, proline, betaine, and phenylalanine, in PTB and PTB–DM patients, consistent with previous reports. Interestingly, there were several organic acids only upregulated in PTB patients or PTB–DM patients, such as aspartate threonine in PTB patients and glycine, xanthine, and guanosine in PTB–DM, which might result from glucose elevation by diabetes. *Lachnospiraceae* and *Ruminococcaceae* within the *Firmicutes* phylum are specialized anaerobic bacteria that produce butyrate, while Bacteroidetes is another major producer of SCFAs, namely acetate and propionate (33, 34). Acetate is a major product of protein degradation and amino acid fermentation within the colon. Under the action of anaerobic intestinal bacteria, amino acids, such as glutamate, histidine, lysine, cysteine, methionine, proline, glycine, and serine, can all be converted into acetate through amino acid deamination (35). In our data, we found that downregulated genera might be responsible for the reduction of these amino acids. More robust downregulation of these genera and metabolites was found in PTB–DM patients, suggesting that DM aggravated the gut microbiota in PTB patients.

Interestingly, it was observed in our result that TMAO was the shared regulated differential metabolite in PTB–DM and PTB patients. The intestinal microbiota metabolizes dietary choline, L-carnitine, and betaine to produce trimethylamine (TMA) and TMAO (36). It has been reported that the abundances of several genera, such as *Ruminococcaceae UCG-009* and *Ruminococcaceae UCG-010*, which belong to the *Firmicutes*, may be related to the reduction in intestinal TMA and plasma TMAO (37). Taken together, the production of TMAO in PTB–DM and PTB patients might be attributed to the reduction of the shared genera, such as *g_Roseburia*, *Ruminococcaceae UCG-013*, and *g_Fimicutes*_*unclassified*, which belong to th*e Firmicutes*. The intestinal microbiota influences bile acid synthesis and biliary cholesterol secretion. Specific serum metabolites in PTB–DM patients, such as cholesterol, might be associated with specific genera regulated individually in PTB–DM patients. Cholesterol, cholic acid, acetone, and indole-3-acetic acid might be potential biomarkers in PTB–DM patients.

However, several limitations exist in this study. First, the specific relationship between gut microbiota and fecal metabolites needs further confirmation through additional experiments, such as fecal microbiota transplantation (FMT). Second, although it is hypothesized that gut microbiota and metabolites, such as amino acids, could serve as biomarkers for PTB and PTB–DM, there is still a lack of sufficient clinical evidence. Third, the number of clinical samples is relatively small, necessitating larger sample studies in the future.

### Conclusion

A comprehensive analysis involving 16S rDNA sequencing, untargeted metabolomics analysis, and the correlation between gut microbiota and metabolites indicates that both PTB and PTB–DM patients exhibit disruptions in the composition and metabolic profile of their regulated gut microbiota. This study contributes to the exploration of key diagnostic biomarkers for PTB and PTB with diabetes. Specifically, shared reductions in the genera, which mostly belong to the *Firmicutes*, such as *g-Ruminococcaceae_UCG*.013 and *g-Ruminococcaceae_NK4A214*, as well as significant regulation of amino acids, such as glycine, serine, and histone, were observed in PTB and PTB–DM patients. Our study extends the understanding of the relationships among gut microbiota, metabolites, PTB, and PTB–DM.

## MATERIALS AND METHODS

### Clinical sample collection

The acquisition of blood samples, stool samples, and clinical data received ethical approval from the Hangzhou Red Cross Hospital Ethics Committee (Approval No. 2020–200). Study participants were divided into three groups: healthy volunteers (Health, *n* = 13), pulmonary tuberculosis patients (PTB, *n* = 13), and pulmonary tuberculosis patients with type 2 diabetes mellitus (PTB–DM, *n* = 13). All the PTB and PTB–DM patients in our study were newly diagnosed cases. None of them had received any anti-tuberculosis antibiotics or other treatments for tuberculosis prior to sample collection. Although most of them had received insulin treatment, their blood glucose levels remained relatively high. The healthy participants in our study had normal lifestyles. In terms of diet, they maintained a balanced diet with a variety of foods. Their sleep patterns were regular, ensuring an adequate amount of sleep every night. They also engaged in regular physical exercise and had effective stress control methods. Moreover, they had no obvious bad habits or addictions, and none of the healthy participants had taken antibiotics within 1 year prior to sampling. Additionally, they had no history of drug abuse. Candidate selection and subject enrollment adhered to predetermined criteria (18), including (i) inclusion criteria for healthy volunteers with physical and fitness; (ii) inclusion criteria for TB patients (1); and (iii) inclusion criteria for type 2 diabetes patients, consisting of individuals presenting typical diabetic symptoms (polyuria, polydipsia, unexplained weight loss), fasting blood glucose ≥11.1 mmol or random blood glucose levels ≥ 11.1 mmol/L, and age between 18 and 65 years. Exclusion criteria included individuals who did not meet inclusion standards or had the following conditions (18): urinary protein quantification exceeding 3.5 g/24 h; primary glomerulonephritis or secondary nephritis unrelated to diabetic nephropathy; urinary protein positivity due to urinary infection; coexisting severe primary disorders, including the heart, brain, lung, liver (alanine aminotransferase [ALT] ≥1.5 times the upper limit of normal), and hematopoietic system disorders; presence of hepatitis, AIDS, rheumatism, or autoimmune diseases; concurrent malignant illnesses; history of diabetic ketoacidosis; poorly controlled hypertension or secondary hypertension; recent occurrence of severe infections within the preceding 2 weeks; pregnancy, allergies; breastfeeding, or intent to become pregnant; participation in other clinical trials within the last 3 months; legally classified disabilities (blindness, deafness, muteness, mental disability, or physical disability); suspected or verified history of alcohol or substance abuse or any circumstance predisposing to missed visits (e.g., frequent changes in work environment); and investigator-assessed potential for poor subject compliance.

### 16S rDNA sequencing

Different DNA extraction kits (OMEGA Serum/Stool DNA Kit) were applied to DNA purification. PCR primers were designed to target the variable region of the 16S rDNA gene or ITS2 rDNA gene against the conserved region. The sequences of 16S rDNA V3–V4 region (464 bp) were amplified by PCR using the universal primer 341F/805R (341F: 5′-CCT ACGGGNGGCWGCAG-3′; 805R: 5′-GACTACHVGGGTATCTAATCC-3′). Following 35 PCR cycles, sequencing adapters and barcodes were added for amplification. Gel electrophoresis was performed to confirm the PCR products, then PCR cleanup and PicoGreen tests were used to purify them. The library was quantified using the Promega QuantiFluor fluorescence quantification instrument. The library was sequenced on the Illumina MiSeq platform, and 250 bp paired-end reads were generated. Finally, the paired-end reads were assigned to each sample by their barcodes and then were assembled to obtain valid raw reads with FLASH software.

### Differential microbiome analysis

The raw reads were quality filtered with fqtrim (v0.94), and chimeric sequences were removed with Vsearch (v2.3.4) to obtain quality-filtered reads. DADA2 was utilized for dereplication as well as the construction of a feature table of unique amplicon sequence variant (ASV). Calculating relative abundance was used for bacterial taxonomy analysis. QIIME2 was used to analyze alpha- and beta-diversity after rarefying the samples to the same sequencing depth for each sample. The visualization of alpha- and beta-diversity, as well as microbiota composition, were performed in R (v3.5.2). Taxonomic biomarkers among groups were determined based on linear discriminant analysis (LDA) scores from LDA effect size (LEfSe) analysis. Blast was used to link ASV annotation sequences with the SILVA databases. Additionally, a random forest analysis at microbial genus levels was conducted to establish the predictive model using the randomForest R package. The data set comprising 39 samples was randomly divided, with 70% allocated for model building, and the remaining 30% used to test the model and performance evaluation. The predictive performance was visualized through non-metric multi-dimensional scaling (NMDS) analysis.

### Untargeted metabolomics

After thawing frozen serum and stool samples on ice, metabolites were extracted using a 50% (v/v) methanol buffer. In brief, 20 µL of each sample was mixed with 120 µL of pre-cooled 50% methanol, followed by 1 min of vortexing and a 10 min room temperature incubation. The extraction mixture was kept at −20°C overnight before being centrifuged for 20 min at 4,000×*g*, 4°C. For LC–MS analysis, the supernatants were transferred to new 96-well plates and kept at −80°C. The quality control (QC) samples were created by combining 10 µL of each extraction combination. An UPLC system with a reversed-phase column was used for the LC–MS analysis. A gradient elution was programmed using a mobile phase consisting of solvent A (water, 0.1% formic acid) and solvent B (acetonitrile, 0.1% formic acid) for chromatographic separation of metabolites. Metabolite detection was performed in both positive and negative ion modes utilizing a high-resolution tandem mass spectrometer, Q-Exactive. IDA mode was used to collect mass spectrometry data. Pretreatments for bioinformatic analysis included peak picking, retention time correction, isotope and adduct annotation, and metabolite annotation based on online databases, such as KEGG and HMDB.

### Differential metabolites analysis

Statistical analyses were performed, followed by untargeted LC–MS/MS data processing, including Student *t*-tests to identify differential metabolites between phenotypes. The resulting *P*-values were adjusted for multiple comparisons using the Benjamini–Hochberg False Discovery Rate (FDR). Supervised orthogonal partial least squares discriminant analysis (OPLS-DA) was carried out using metaX to discriminate between different variables among groups. The variable importance in projection (VIP) value was calculated, and a VIP cutoff of 1.0 was used to select important metabolite features.

### Deconfounding analysis of omics data

Due to the limited sample size in the omics studies, covariate deconfounding was implemented to identify an unbiased and unconfounded correlation between omics features (differential genus, differential fecal metabolites, and differential serum metabolites) and individual health statuses (health, PTB, or PTB–DM) in addition to variables, such as BMI, HbA1c, blood sugar levels, and other biochemical compounds outlined in Table S1 (18). The univariate analysis was conducted using metadeconfoundR (version 1.0.2), where the relative abundances or intensities of omics features were evaluated for associations with each clinical variable. These associations required a Benjamini–Hochberg adjusted false discovery rate (FDR) of <0.1 and the absence of any evident confounders. Non-parametric tests were used for all association analyses to reduce reliance on assumptions regarding feature distributions. The Mann–Whitney test was used for two categorical variables, and non-parametric Spearman correlation tests were applied for pairs of continuous variables. In summary, when a feature exhibits a significant relationship with at least two covariates, these covariates may be categorized as strictly deconfounded, ambiguously deconfounded, or confounded.

### Statistical analysis

The statistical significance of each difference was analyzed by using an unpaired Student’s *t*-test or one-way ANOVA followed by Tukey’s post hoc tests. A *P* < 0.05 was considered statistically significant. Data are shown as the mean ± SEM (GraphPad Prism 6).

## Data Availability

Raw sequencing data of all 16S rRNA sequences have been deposited into the NCBI Sequence Read Archive (SRA) under accession number PRJNA1026066. Serum and fecal metabolomics raw data are uploaded to MetaboLights under project accession numbers MTBLS7623 and MTBLS8729.

## AFTER EPUB

[This article was published on 14 March 2025 with errors in the text and supplemental file. The errors were corrected in the current version, posted on 14 July 2025.]

## SUPPLEMENTAL MATERIAL

Figure S1 spectrum.01772-24-s0001.TIF Representative lung CT images from the Health, PTB, and PTB–DM groups.

Figure S2 spectrum.01772-24-s0002.tif Prediction of PTB and PTB–DM using random forest-based machine learning.

Figure S3 spectrum.01772-24-s0003.tif The interplay between gut microbiota and fecal metabolome was shown, with different colored blocks representing PTB, PTB–DM, and shared alterations in microbial genera and metabolites between them.

Figure S4 spectrum.01772-24-s0004.tif Clustering community types (or enterotypes) at genus level based on the Dirichlet multinomial model.

Figure S5 spectrum.01772-24-s0005.tif Serum metabolome was visualized, with different colored blocks representing PTB, PTB–DM, and shared alterations in microbial genera and metabolites between them.

Supplemental material spectrum.01772-24-s0006.docx Supplemental figure legends and Table S1.

Table S2 spectrum.01772-24-s0007.docx Differential metabolites of the fecal metabolome.

Table S3 spectrum.01772-24-s0008.docx Pathway enrichment of fecal differential metabolites.

Table S4 spectrum.01772-24-s0009.docx Differential metabolites of serum metabolome.

Table S5 spectrum.01772-24-s0010.docx Pathway enrichment of serum differential metabolites.

ASM does not own the copyrights to Supplemental Material that may be linked to, or accessed through, an article. The authors have granted ASM a non-exclusive, world-wide license to publish the Supplemental Material files. Please contact the corresponding author directly for reuse.
