# Supplementary figures and images for: Disorders of gut microbiota and fecal–serum metabolic patterns are associated with pulmonary tuberculosis and pulmonary tuberculosis comorbid type 2 diabetes mellitus
Source: Microbiol Spectr. 2025 Mar 14;13(8):e01772-24. doi: 10.1128/spectrum.01772-24 (PMC12323600; doi:10.1128/spectrum.01772-24)

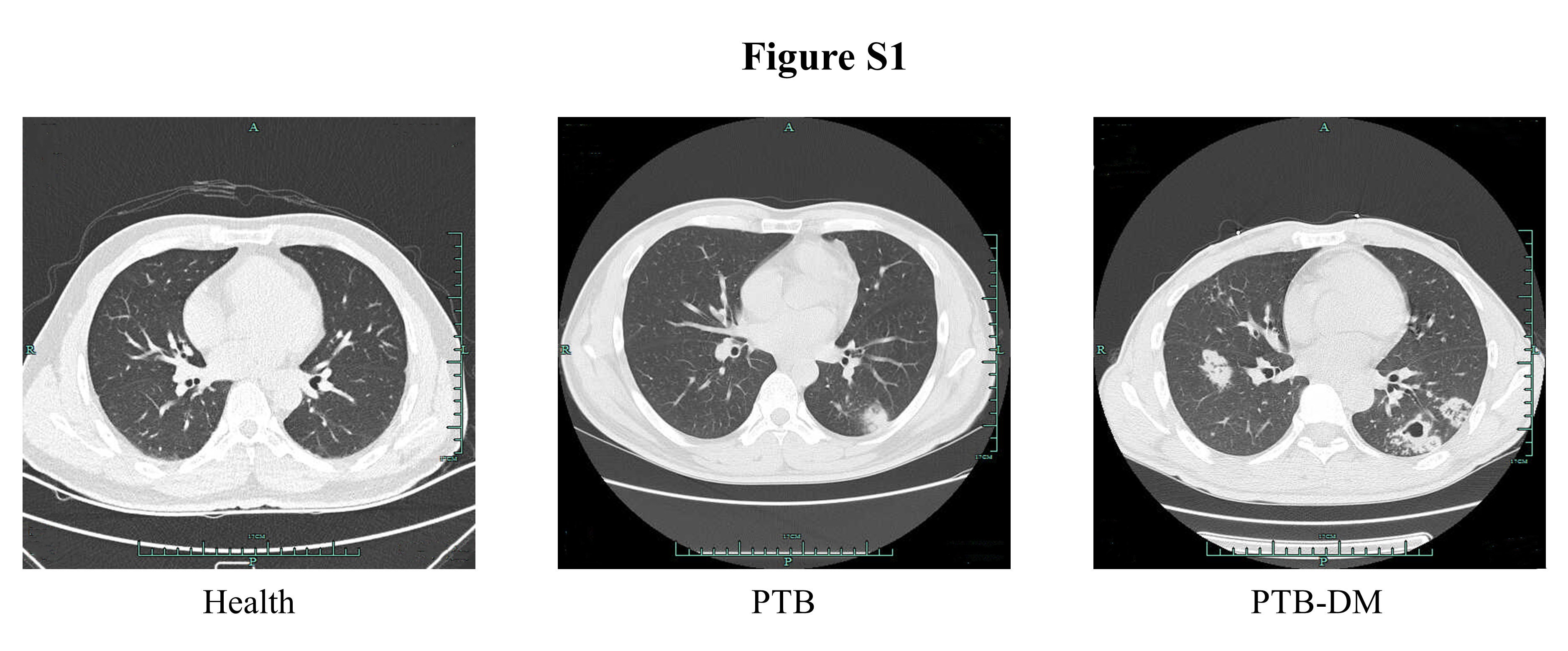

Supplement: Figure S1 — Representative lung CT images from the Health, PTB, and PTB–DM groups. [file spectrum.01772-24-s0001.tif]

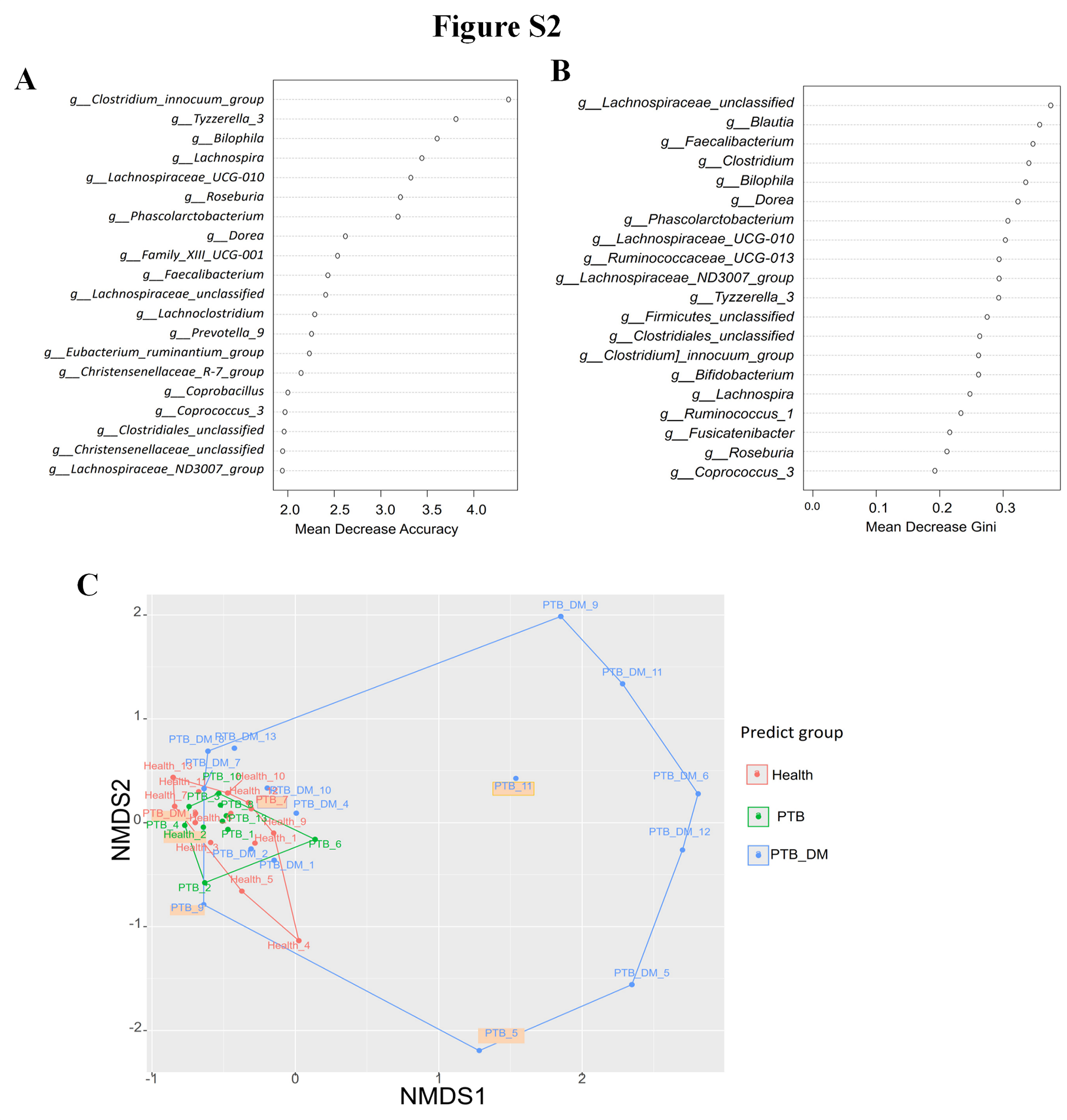

Supplement: Figure S2 — Prediction of PTB and PTB–DM using random forest-based machine learning. [file spectrum.01772-24-s0002.tif]

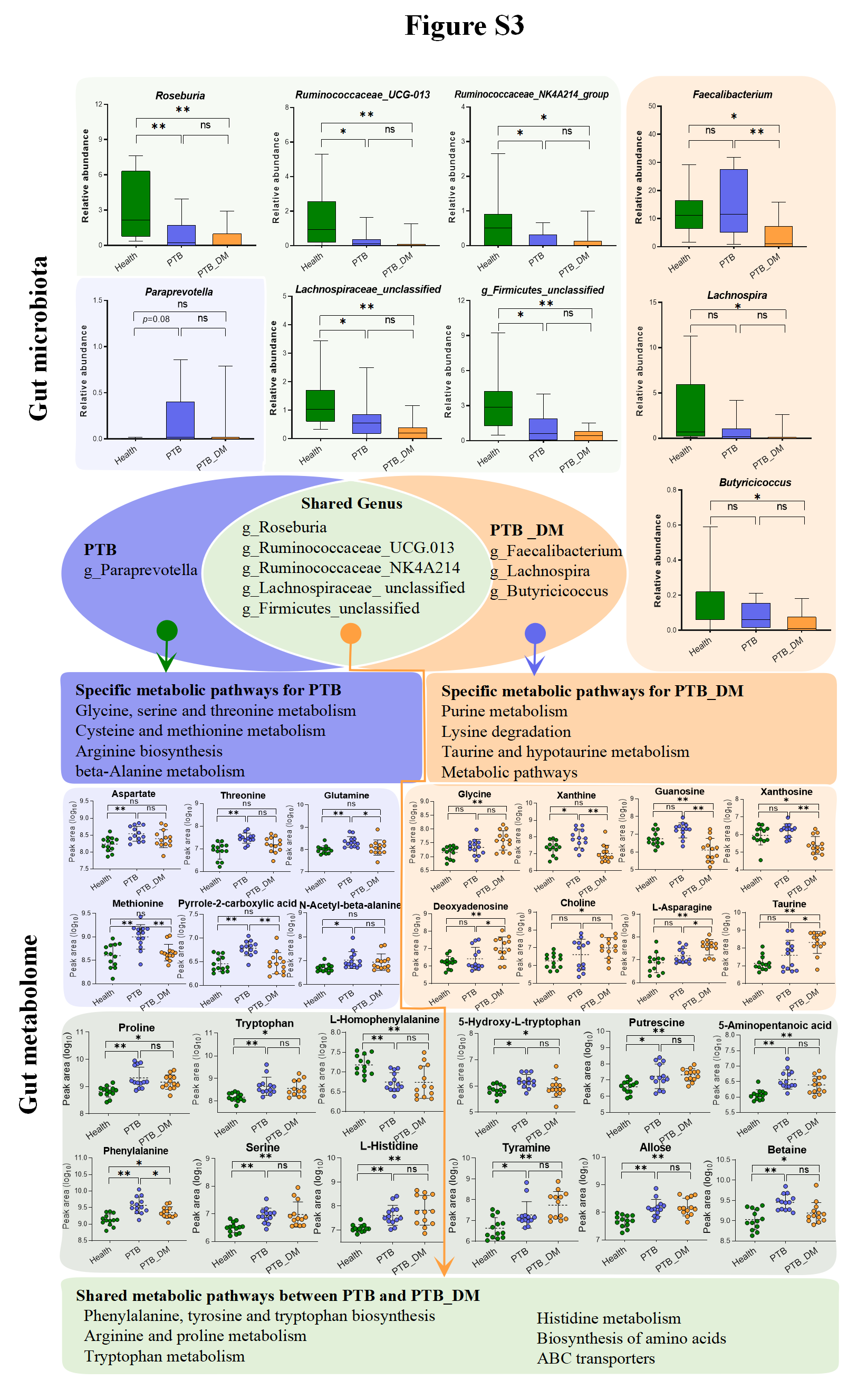

Supplement: Figure S3 — The interplay between gut microbiota and fecal metabolome was shown, with different colored blocks representing PTB, PTB–DM, and shared alterations in microbial genera and metabolites between them. [file spectrum.01772-24-s0003.tif]

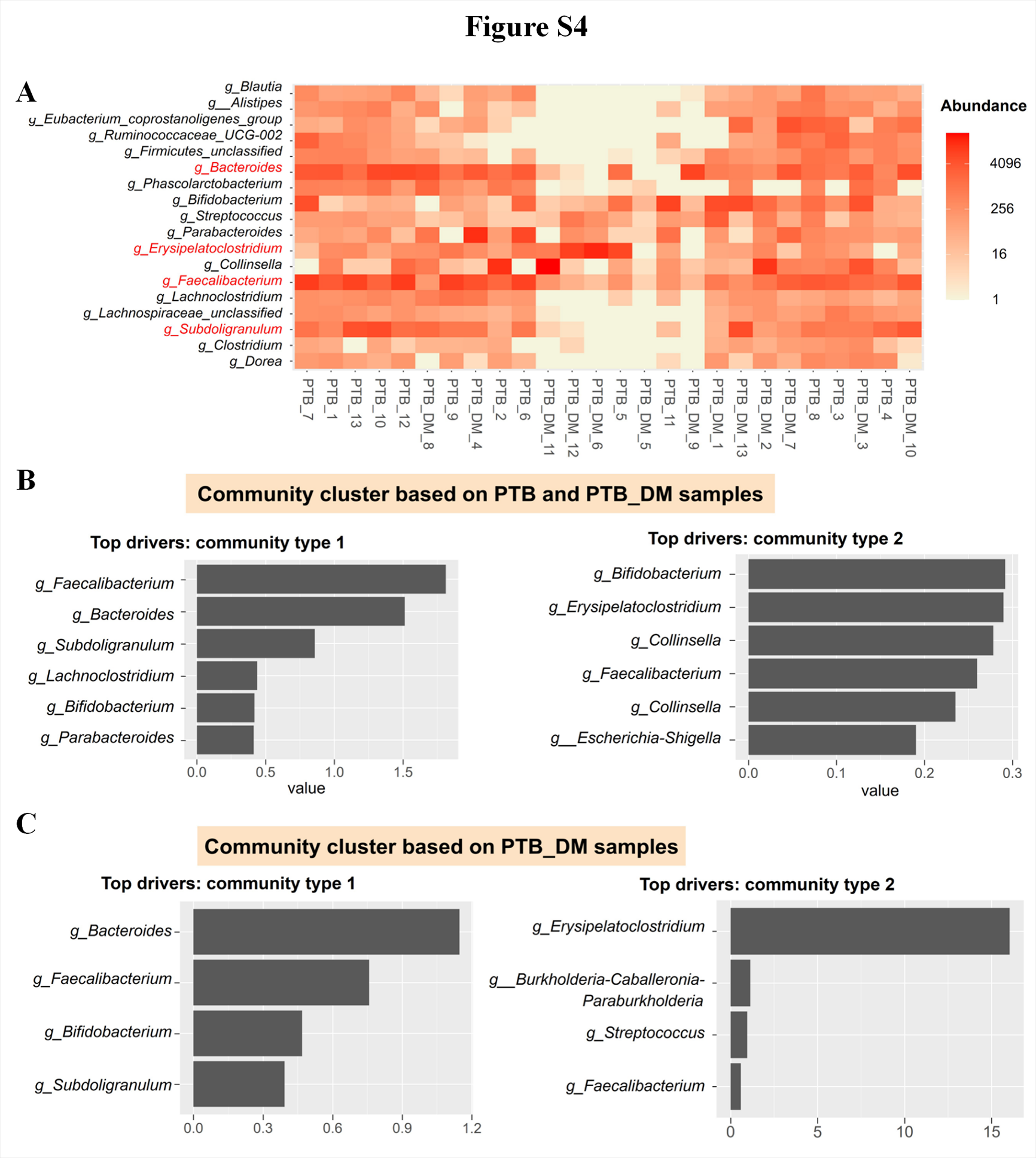

Supplement: Figure S4 — Clustering community types (or enterotypes) at genus level based on the Dirichlet multinomial model. [file spectrum.01772-24-s0004.tif]

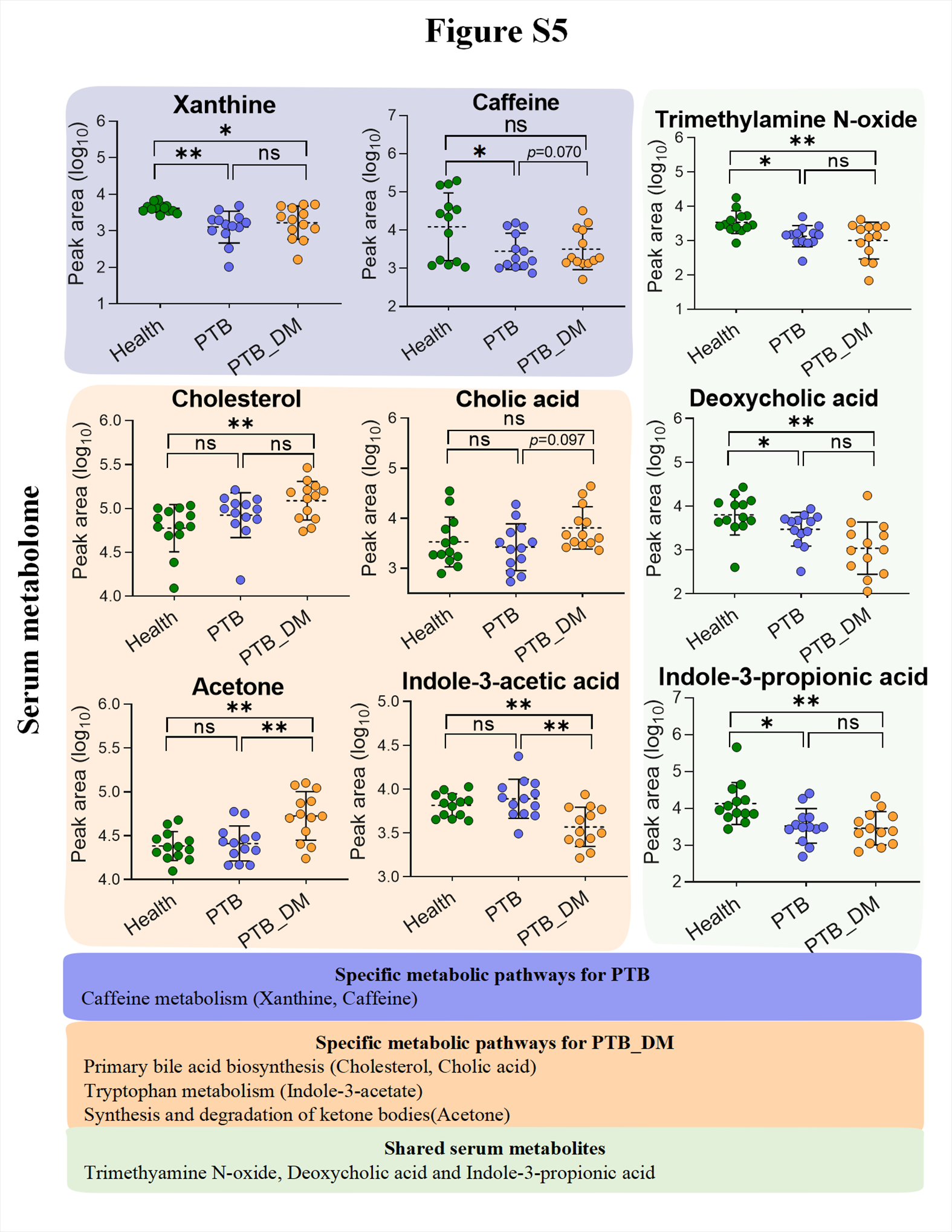

Supplement: Figure S5 — Serum metabolome was visualized, with different colored blocks representing PTB, PTB–DM, and shared alterations in microbial genera and metabolites between them. [file spectrum.01772-24-s0005.tif]
