# Supplementary material for: Disorders of gut microbiota and fecal–serum metabolic patterns are associated with pulmonary tuberculosis and pulmonary tuberculosis comorbid type 2 diabetes mellitus
Source: Microbiol Spectr. 2025 Mar 14;13(8):e01772-24. doi: 10.1128/spectrum.01772-24 (PMC12323600; doi:10.1128/spectrum.01772-24)
Supplement: Supplemental material — Supplemental figure legends and Table S1. [file spectrum.01772-24-s0006.docx]

**Supplementary figure legends**

**Figure S1. Representative lung CT images from the** **Health, PTB, and PTB-DM groups.**

**Figure S2. Prediction of PTB and PTB_BB using random forest-based machine learning.** The up panel shows the importance of genus features based on mean decrease in prediction accuracy (A) and Gini indexes (B). The selected 20 genus features were utilized to construct a refined model and the prediction results was visualized by non-metric multi-dimensional scaling (NMDS) analysis. In the NMDS plot (C), the orange background color represents the incorrect prediction, including Health_2, PTB_5, PTB_9, PTB_11, and PTB_DM_3 samples.

**Figure S3. The interplay between gut microbiota and fecal metabolome was shown, with different colored blocks representing PTB, PTB-DM, and shared alterations in microbial genera and metabolites between them.**

**Figure S4 Clustering community types (or enterotypes) at genus level based on the Dirichlet multinomial model.** Heatmap shows the abundance of 18 core genus that are prevalent at 0.1% relative abundance in 50% of the samples (A). Bar plots show the relative contribution of each core genus to each community type, and the x axis displays the cluster importance for each core genus.

**Figure S5. Serum metabolome was visulaized, with different colored blocks representing PTB, PTB-DM, and shared alterations in microbial genera and metabolites between them.**

**Table S1 Clinical data of the candidates included in this study as our previous research reported(1)**

| **Medical index Group** | **Health（n=13）** | **PTB**  **(n=13)** | **PTB_DM**  **(n=13)** |
| --- | --- | --- | --- |
| Age (years) | 40.2±6.6 | 31.4±11.0 | 50.9±11.9 |
| Gender (male/female) | (4/9) | (10/3) | (11/2) |
| BMI (kg/m^2^) | 23.7±3.1 | 19.6±2.3 | 23.9±2.7 |
| Hypertension (+/-) | (0/13) | (0/13) | (0/13) |
| Diabetes (+/-) | (0/13) | (0/13) | (13/0)^##^ ** |
| Tuberculin skin test (+/-) | (0/13) | (11/2)^##^ | (13/0)^##^ |
| γ interferon gamma release assay (+/-) | (0/13) | (10/3)^##^ | (13/0)^##^ |
| GeneXpert MTB/RIF (+/-) | (0/13) | (9/4)^##^ | (13/0)^##^ * |
| Imaging features of tuberculosis -Lesion (+/-) | (0/13) | (13/0)^##^ | (13/0)^##^ ** |
| Sputum culture positive (+/-) | (0/13) | (9/4)^##^ | (13/0)^##^ * |
| Sputum smear positive (+/-) | (0/13) | (7/6)^##^ | (13/0)^##^ |
| Cough > 2 weeks (+/-) | (0/13) | (12/1)^##^ | (12/1)^##^ |
| Fever > 2 weeks (+/-) | (0/13) | (1/12) | (2/11)^##^ |
| Night sweats > 2 weeks (+/-) | (0/13) | (0/13) | (2/11) |
| Past history of tuberculosis (+/-) | (0/13) | (0/13) | (0/13) |
| Glyeosylated hemoglobin A1c (%) | 4.9±0.7 | 4.4±0.7 | 10±2.6^##^ ** |
| [Blood glucose](https://fanyi.so.com/?src=onebox# blood glucose) (mmol/L) | 5.3±0.4 | 5.7±1.1 | 12.4±8.2^##^ ** |
| Total protein (g/L) | 75.5±3.1 | 72.2±7.0 | 71.9±6.7 |
| White blood cell (10^9^/L) | 6.1±1.6 | 6.7±1.6 | 6.0±2.6 |
| Red blood cell (10^12^/L) | 4.9±0.7 | 4.6±0.4 | 4.6±0.8 |
| Hemoglobin (g/L) | 134.4±16.1 | 138.1±14.2 | 129.5±19.7 |
| Platelet (10^9^/L) | 279.2±82.4 | 279.5±100.8 | 233.4±67.8 |
| [Creatinine](file:///C:/Program%2520Files%2520(x86)/youdao/dict/Application/8.9.9.0/resultui/html/index.html#/javascript:;) (μmol/L) | 75.21±11.1 | 76.2±15.3 | 80.5±18.5 |
| [High density lipoprotein](https://www.so.com/link?m=bGdxAuDROaT7qfQGMDMd8bE3RAbBYUVX7IhOAtxbuT4ljEqXZEcEqqg19GAguEsEt92QNJ+O+7kn62mwCJlkIPxj+KV2IUJ+PDna1Y7j4rJK//npU5/3DJAWqkERg1SV2iHczLT1wWOohiyOqzZtNW2hDr/IY4YgnOaqFrQ==) (mmol/L) | 1.5±0.3 | 1.3±0.8 | 1.0±0.1 |
| [Low density lipoprotein](https://www.so.com/link?m=bGdxAuDROaT7qfQGMDMd8bE3RAbBYUVX7IhOAtxbuT4ljEqXZEcEqqg19GAguEsEt92QNJ+O+7kn62mwCJlkIPxj+KV2IUJ+PDna1Y7j4rJK//npU5/3DJAWqkERg1SV2iHczLT1wWOohiyOqzZtNW2hDr/IY4YgnOaqFrQ==) (mmol/L) | 2.84±0.6 | 2.4±0.6 | 2.8±0.5 |

^##^*p*<0.01 VS.Health ; *,*p*<0.05 VS.PTB ;**,*p*<0.01 VS.PTB

1. Wang Y, He X, Zheng D, He Q, Sun L, Jin J. 2023. Integration of Metabolomics and Transcriptomics Reveals Major Metabolic Pathways and Potential Biomarkers Involved in Pulmonary Tuberculosis and Pulmonary Tuberculosis-Complicated Diabetes. Microbiol Spectr 11:e0057723.
