# Supplementary material for: Disorders of gut microbiota and fecal–serum metabolic patterns are associated with pulmonary tuberculosis and pulmonary tuberculosis comorbid type 2 diabetes mellitus
Source: Microbiol Spectr. 2025 Mar 14;13(8):e01772-24. doi: 10.1128/spectrum.01772-24 (PMC12323600; doi:10.1128/spectrum.01772-24)
Supplement: Table S2 — Differential metabolites of the fecal metabolome. [file spectrum.01772-24-s0007.docx]

**Table S2 Differential metabolites of fecal metabolome**

| **PTB VS Health.significant** | | | | | | |
| --- | --- | --- | --- | --- | --- | --- |
| **ID** | **MS2superclass** | **MS2Metabolite** | **MS2kegg** | **VIP** | **FC** | **wilcox.test_**  **p.value_BHcorrect** |
| neg-M507T256 | Lipids and lipid-like molecules | LysoPG 18:2 | C05980 | 2.391 | 0.285 | 0.004 |
| neg-M403T249 | Lipids and lipid-like molecules | Dihydroxy-3-oxo-4-cholenoic acid | C15568 | 1.698 | 0.419 | 0.008 |
| pos-M454T334 | Lipids and lipid-like molecules | Acylcarnitine 20:1 | C02301 | 2.359 | 4.780 | 0.010 |
| neg-M519T264 | Lipids and lipid-like molecules | LysoPA 24:1 | C00681 | 1.717 | 0.337 | 0.011 |
| neg-M204T76 | Lipids and lipid-like molecules | 5-Acetamidovalerate | C03087 | 3.039 | 5.987 | 0.013 |
| neg-M161T82 | Lipids and lipid-like molecules | 3-Hydroxymethylglutaric acid | C03761 | 2.426 | 10.470 | 0.015 |
| pos-M118T85 | Lipids and lipid-like molecules | L-Norvaline | C01826 | 2.204 | 3.608 | 0.017 |
| pos-M297T261 | Lipids and lipid-like molecules | 9,10-Epoxyoctadecenoic acid | C14825 | 2.417 | 0.194 | 0.021 |
| neg-M345T227_2 | Lipids and lipid-like molecules | Gibberellic acid | C01699 | 1.448 | 0.468 | 0.023 |
| pos-M495T259 | Lipids and lipid-like molecules | Betulinic acid | C08619 | 2.272 | 0.262 | 0.023 |
| neg-M315T317 | Lipids and lipid-like molecules | 9,10-dihydroxyoctadecanoate | C15988 | 2.039 | 0.297 | 0.030 |
| pos-M315T261 | Lipids and lipid-like molecules | Oxylipin | C14829 | 2.612 | 0.163 | 0.038 |
| neg-M227T221 | Lipids and lipid-like molecules | Traumatic acid | C16308 | 1.949 | 0.308 | 0.038 |
| pos-M445T240 | Lipids and lipid-like molecules | Bufotalin | C16923 | 2.917 | 0.027 | 0.038 |
| pos-M235T213 | Lipids and lipid-like molecules | Valerenic acid | C09743 | 2.116 | 0.182 | 0.043 |
| neg-M187T190 | Organic acids and derivatives | Gly-Leu | C02155 | 1.689 | 2.458 | 0.002 |
| pos-M120T84 | Organic acids and derivatives | Threonine | C00188 | 2.380 | 3.172 | 0.005 |
| neg-M88T193 | Organic acids and derivatives | 2-Aminopropanoic acid | C01401 | 1.874 | 2.051 | 0.005 |
| neg-M190T206 | Organic acids and derivatives | N-Acetyl-L-methionine | C02712 | 2.370 | 4.063 | 0.005 |
| pos-M166T186_2 | Organic acids and derivatives | Phenylalanine | C00079 | 2.306 | 2.981 | 0.005 |
| neg-M116T74 | Organic acids and derivatives | 5-Aminopentanoic acid | C00431 | 2.269 | 3.802 | 0.005 |
| neg-M267T84 | Organic acids and derivatives | His-Leu | C05010 | 2.120 | 3.160 | 0.009 |
| neg-M161T45 | Organic acids and derivatives | Diethyl dicarbonate | C11592 | 1.587 | 2.341 | 0.009 |
| pos-M147T46 | Organic acids and derivatives | Glutamine | C00064 | 1.644 | 2.396 | 0.010 |
| pos-M134T53 | Organic acids and derivatives | D-Aspartic acid | C00402 | 2.001 | 2.616 | 0.010 |
| neg-M130T191 | Organic acids and derivatives | Beta-Leucine | C02486 | 1.957 | 2.499 | 0.010 |
| pos-M106T72 | Organic acids and derivatives | Serine | C00065 | 1.895 | 3.000 | 0.011 |
| neg-M154T44 | Organic acids and derivatives | L-Histidine | C00135 | 2.028 | 5.166 | 0.011 |
| neg-M176T201 | Organic acids and derivatives | N-Formyl-L-Methionine | C03145 | 2.048 | 4.442 | 0.013 |
| neg-M181T75 | Organic acids and derivatives | 4-Hydroxyisophthalic acid | C21168 | 2.081 | 3.591 | 0.015 |
| pos-M132T104_1 | Organic acids and derivatives | Isoleucine | C00407 | 2.010 | 2.978 | 0.017 |
| pos-M118T54_2 | Organic acids and derivatives | Betaine | C00719 | 2.120 | 2.525 | 0.017 |
| pos-M227T45 | Organic acids and derivatives | Carnosine | C00386 | 1.799 | 2.448 | 0.018 |
| pos-M180T189_2 | Organic acids and derivatives | L-Homophenylalanine | C17235 | 1.947 | 0.366 | 0.018 |
| neg-M423T248 | Organic acids and derivatives | Pravastatin | C01844 | 2.360 | 0.306 | 0.021 |
| pos-M150T73_2 | Organic acids and derivatives | Methionine | C00073 | 2.010 | 2.521 | 0.023 |
| pos-M132T186 | Organic acids and derivatives | trans-4-Hydroxy-L-proline | C01157 | 1.909 | 2.188 | 0.023 |
| neg-M130T96 | Organic acids and derivatives | N-Acetyl-beta-alanine | C01073 | 1.579 | 3.086 | 0.026 |
| neg-M132T46 | Organic acids and derivatives | Aspartate | C00049 | 1.543 | 2.046 | 0.048 |
| neg-M212T218 | Organic acids and derivatives | alpha-Kainic acid | C12819 | 2.182 | 13.179 | 0.048 |
| neg-M182T201 | Organoheterocyclic compounds | Saccharin | C12283 | 3.622 | 0.032 | 0.001 |
| pos-M226T192 | Organoheterocyclic compounds | 6-Benzylaminopurine | C11263 | 2.768 | 0.085 | 0.004 |
| pos-M136T190 | Organoheterocyclic compounds | Adenine | C00147 | 1.621 | 2.079 | 0.005 |
| pos-M205T198 | Organoheterocyclic compounds | Tryptophan | C00078 | 2.174 | 4.840 | 0.007 |
| neg-M110T83 | Organoheterocyclic compounds | Pyrrole-2-carboxylic acid | C05942 | 1.708 | 2.107 | 0.007 |
| neg-M148T228 | Organoheterocyclic compounds | 5,6-Dihydroxyindole | C05578 | 2.391 | 6.210 | 0.009 |
| neg-M375T217 | Organoheterocyclic compounds | Riboflavin | C00255 | 2.522 | 0.210 | 0.010 |
| pos-M166T84 | Organoheterocyclic compounds | 3-Methylguanine | C02230 | 2.215 | 3.474 | 0.013 |
| pos-M118T186 | Organoheterocyclic compounds | Indole | C00463 | 2.002 | 2.604 | 0.017 |
| pos-M132T191_2 | Organoheterocyclic compounds | 3-Methylindole | C08313 | 1.447 | 2.029 | 0.018 |
| pos-M70T51 | Organoheterocyclic compounds | 1-Pyrroline | C15668 | 2.249 | 4.364 | 0.018 |
| neg-M138T48_2 | Organoheterocyclic compounds | 6-Hydroxynicotinic acid | C01020 | 2.152 | 0.239 | 0.021 |
| pos-M86T104_2 | Organoheterocyclic compounds | Piperidine | C01746 | 2.060 | 2.863 | 0.021 |
| pos-M192T218 | Organoheterocyclic compounds | 5-Hydroxyindole-3-acetic acid | C05635 | 2.332 | 0.136 | 0.023 |
| neg-M219T86 | Organoheterocyclic compounds | 5-Hydroxy-L-tryptophan | C00643 | 1.584 | 2.324 | 0.026 |
| pos-M323T216 | Organoheterocyclic compounds | Mequitazine | C12755 | 1.488 | 2.652 | 0.030 |
| neg-M165T228 | Phenylpropanoids and polyketides | L-3-Phenyllactic acid | C05607 | 2.883 | 7.134 | 0.002 |
| neg-M285T279 | Phenylpropanoids and polyketides | Isosakuranetin | C05334 | 3.984 | 89.547 | 0.002 |
| neg-M135T242 | Phenylpropanoids and polyketides | Caffeic acid | C01481 | 1.344 | 0.497 | 0.003 |
| neg-M271T251 | Phenylpropanoids and polyketides | Naringenin | C00509 | 3.606 | 39.365 | 0.008 |
| neg-M147T228 | Phenylpropanoids and polyketides | Cinnamic acid | C10438 | 2.478 | 6.318 | 0.009 |
| pos-M165T84 | Phenylpropanoids and polyketides | 2-Coumaric acid | C01772 | 2.079 | 3.239 | 0.015 |
| pos-M147T198 | Phenylpropanoids and polyketides | Coumarin | C05851 | 1.736 | 3.845 | 0.021 |
| neg-M579T218_1 | Phenylpropanoids and polyketides | Naringin | C09789 | 3.542 | 123.214 | 0.021 |
| pos-M285T241 | Phenylpropanoids and polyketides | Biochanin A | C00814 | 1.703 | 3.880 | 0.048 |
| neg-M253T239 | Phenylpropanoids and polyketides | Daidzein | C10208 | 2.114 | 3.202 | 0.048 |
| neg-M119T227 | Benzenoids | 2-Methylbenzaldehyde | C07214 | 2.831 | 7.200 | 0.002 |
| neg-M135T225 | Benzenoids | M-toluic Acid | C07211 | 2.227 | 0.201 | 0.008 |
| neg-M218T192 | Benzenoids | N-Phenyl-2-naphthylamine | C14694 | 1.850 | 2.022 | 0.010 |
| pos-M136T84 | Benzenoids | 2-Phenylacetamide | C02505 | 2.068 | 3.200 | 0.015 |
| pos-M325T196_2 | Benzenoids | Citalopram | C07572 | 2.522 | 0.122 | 0.015 |
| neg-M191T46 | Organic oxygen compounds | D-(-)-Quinic acid | C06746 | 1.553 | 2.358 | 0.011 |
| pos-M123T85 | Organic oxygen compounds | 4-Hydroxybenzaldehyde | C00633 | 1.987 | 3.039 | 0.017 |
| neg-M163T74_3 | Organic oxygen compounds | L-Fucose | C01019 | 1.998 | 4.429 | 0.048 |
| pos-M284T104 | Nucleosides, nucleotides, and analogues | Guanosine | C00387 | 1.857 | 2.615 | 0.038 |
| pos-M133T51 | Amino Acids | DL-Ornithine | C01602 | 3.295 | 5.855 | 0.013 |
| neg-M179T52 | Carbohydrates | Allose | C01487 | 2.077 | 3.510 | 0.010 |
| **PTB_DM VS Health.significant** | | | | | | |
| **ID** | **MS2superclass** | **MS2Metabolite** | **MS2kegg** | **VIP** | **FC** | **wilcox.test_**  **p.value_BHcorrect** |
| neg-M187T232 | Lipids and lipid-like molecules | Azelaic acid | C08261 | 2.152 | 0.356 | 0.001 |
| neg-M487T286 | Lipids and lipid-like molecules | Ananasic acid | C12599 | 1.919 | 0.159 | 0.001 |
| pos-M358T265 | Lipids and lipid-like molecules | Acylcarnitine 13:0 | C02301 | 2.996 | 59.250 | 0.006 |
| neg-M173T222 | Lipids and lipid-like molecules | Suberic acid | C08278 | 1.769 | 0.427 | 0.006 |
| pos-M495T259 | Lipids and lipid-like molecules | Betulinic acid | C08619 | 2.185 | 0.196 | 0.006 |
| pos-M445T240 | Lipids and lipid-like molecules | Bufotalin | C16923 | 3.035 | 0.012 | 0.007 |
| neg-M351T267 | Lipids and lipid-like molecules | Thromboxane A2 | C02198 | 2.273 | 0.224 | 0.008 |
| pos-M503T366 | Lipids and lipid-like molecules | GW7647 | C15622 | 1.707 | 0.316 | 0.008 |
| neg-M129T79 | Lipids and lipid-like molecules | Citraconic acid | C02226 | 2.053 | 3.840 | 0.010 |
| pos-M400T301 | Lipids and lipid-like molecules | Palmitoylcarnitine | C02990 | 2.591 | 11.299 | 0.010 |
| neg-M453T325 | Lipids and lipid-like molecules | LysoPG 14:1 | C05980 | 3.007 | 4.655 | 0.011 |
| pos-M293T248 | Lipids and lipid-like molecules | Colnelenic acid | C16320 | 1.807 | 0.336 | 0.012 |
| neg-M942T260 | Lipids and lipid-like molecules | Soyasaponin Bb | C08983 | 2.613 | 31.766 | 0.017 |
| pos-M301T250 | Lipids and lipid-like molecules | Retinoic acid | C00777 | 1.496 | 3.214 | 0.019 |
| neg-M161T82 | Lipids and lipid-like molecules | 3-Hydroxymethylglutaric acid | C03761 | 2.198 | 7.842 | 0.019 |
| neg-M229T260 | Lipids and lipid-like molecules | Dodecanedioic acid | C02678 | 2.719 | 0.209 | 0.022 |
| neg-M549T406 | Lipids and lipid-like molecules | MGDG 15:1 | C03692 | 2.666 | 14.136 | 0.022 |
| pos-M301T360 | Lipids and lipid-like molecules | Allylestrenol | C12811 | 2.143 | 0.164 | 0.022 |
| neg-M528T272 | Lipids and lipid-like molecules | Glycochenodeoxycholic acid 7-sulfate | C15559 | 2.805 | 20.610 | 0.022 |
| neg-M345T233_1 | Lipids and lipid-like molecules | Gibberellic acid | C01699 | 2.166 | 0.146 | 0.032 |
| neg-M395T316 | Lipids and lipid-like molecules | Pregnenolone sulfate | C18044 | 2.938 | 8.783 | 0.032 |
| neg-M175T75 | Lipids and lipid-like molecules | 2-Isopropylmalic acid | C02504 | 2.219 | 20.466 | 0.035 |
| neg-M201T242_1 | Lipids and lipid-like molecules | Sebacic acid | C08277 | 2.126 | 0.373 | 0.035 |
| neg-M227T254 | Lipids and lipid-like molecules | Traumatic acid | C16308 | 2.336 | 0.368 | 0.035 |
| neg-M365T262 | Lipids and lipid-like molecules | 20-Carboxy-leukotriene B4 | C05950 | 1.697 | 0.467 | 0.040 |
| neg-M479T395_2 | Lipids and lipid-like molecules | Brassinolide | C11049 | 2.093 | 0.258 | 0.040 |
| neg-M315T344 | Lipids and lipid-like molecules | 9,10-dihydroxyoctadecanoate | C15988 | 2.197 | 65.799 | 0.040 |
| neg-M331T261 | Lipids and lipid-like molecules | Floionolic acid | C19621 | 2.297 | 0.191 | 0.040 |
| pos-M197T221 | Lipids and lipid-like molecules | Cantharidin | C16778 | 1.552 | 0.387 | 0.040 |
| pos-M407T302 | Lipids and lipid-like molecules | Zymosterol | C05437 | 1.610 | 0.201 | 0.044 |
| pos-M347T247 | Lipids and lipid-like molecules | Corticosterone | C02140 | 1.537 | 0.439 | 0.049 |
| neg-M403T249 | Lipids and lipid-like molecules | Dihydroxy-3-oxo-4-cholenoic acid | C15568 | 1.720 | 0.455 | 0.049 |
| pos-M106T72 | Organic acids and derivatives | Serine | C00065 | 1.686 | 5.107 | 0.010 |
| neg-M124T46 | Organic acids and derivatives | Taurine | C00245 | 2.722 | 15.547 | 0.010 |
| pos-M130T78 | Organic acids and derivatives | L-5-Oxoproline | C01879 | 1.781 | 3.778 | 0.010 |
| pos-M132T198_2 | Organic acids and derivatives | trans-4-Hydroxy-L-proline | C01157 | 1.871 | 3.553 | 0.012 |
| neg-M154T44 | Organic acids and derivatives | L-Histidine | C00135 | 2.285 | 11.480 | 0.015 |
| pos-M116T52 | Organic acids and derivatives | Proline | C00148 | 1.285 | 2.413 | 0.019 |
| neg-M131T161 | Organic acids and derivatives | Glutaric acid | C00489 | 2.145 | 0.298 | 0.019 |
| neg-M131T47 | Organic acids and derivatives | L-Asparagine | C00152 | 2.199 | 3.646 | 0.024 |
| pos-M134T53 | Organic acids and derivatives | D-Aspartic acid | C00402 | 1.386 | 2.220 | 0.024 |
| neg-M116T74 | Organic acids and derivatives | 5-Aminopentanoic acid | C00431 | 1.277 | 2.335 | 0.028 |
| pos-M172T51 | Organic acids and derivatives | Tetrahydrodipicolinate | C03972 | 1.376 | 0.339 | 0.028 |
| pos-M277T51 | Organic acids and derivatives | L-Saccharopine | C00449 | 1.613 | 0.369 | 0.032 |
| pos-M227T45 | Organic acids and derivatives | Carnosine | C00386 | 1.525 | 3.165 | 0.035 |
| neg-M181T75 | Organic acids and derivatives | 4-Hydroxyisophthalic acid | C21168 | 1.978 | 3.845 | 0.040 |
| pos-M76T45 | Organic acids and derivatives | Glycine | C00037 | 1.598 | 3.293 | 0.044 |
| neg-M138T48_2 | Organoheterocyclic compounds | 6-Hydroxynicotinic acid | C01020 | 2.364 | 0.157 | 0.003 |
| neg-M183T50 | Organoheterocyclic compounds | Thiouric acid | C16613 | 2.060 | 0.283 | 0.003 |
| neg-M148T228 | Organoheterocyclic compounds | 5,6-Dihydroxyindole | C05578 | 2.475 | 11.028 | 0.003 |
| pos-M226T192 | Organoheterocyclic compounds | 6-Benzylaminopurine | C11263 | 2.516 | 0.083 | 0.006 |
| pos-M204T46 | Organoheterocyclic compounds | 3-Indolebutyric acid | C11284 | 1.911 | 0.275 | 0.008 |
| pos-M219T218 | Organoheterocyclic compounds | N-Acetyl-5-hydroxytryptamine | C00978 | 2.136 | 22.979 | 0.011 |
| neg-M182T201 | Organoheterocyclic compounds | Saccharin | C12283 | 1.628 | 0.084 | 0.012 |
| neg-M111T54_1 | Organoheterocyclic compounds | 2-Furoic acid | C01546 | 2.300 | 10.652 | 0.017 |
| neg-M155T44 | Organoheterocyclic compounds | Imidazole-4-acetaldehyde | C05130 | 2.220 | 12.225 | 0.017 |
| pos-M177T183 | Organoheterocyclic compounds | Serotonin | C00780 | 1.895 | 3.285 | 0.019 |
| pos-M70T51 | Organoheterocyclic compounds | 1-Pyrroline | C15668 | 1.185 | 2.199 | 0.019 |
| pos-M205T198 | Organoheterocyclic compounds | Tryptophan | C00078 | 1.430 | 3.209 | 0.022 |
| pos-M169T187 | Organoheterocyclic compounds | Pyridoxamine | C00534 | 1.482 | 0.417 | 0.022 |
| pos-M135T204 | Organoheterocyclic compounds | 1-Isobenzofuranone | C09921 | 1.386 | 5.064 | 0.024 |
| neg-M190T219 | Organoheterocyclic compounds | 5-Hydroxyindole-3-acetic acid | C05635 | 2.330 | 0.118 | 0.049 |
| neg-M579T218_1 | Phenylpropanoids and polyketides | Naringin | C09789 | 2.796 | 108.589 | 0.001 |
| neg-M593T203 | Phenylpropanoids and polyketides | Vicenin 2 | C10195 | 2.444 | 25.405 | 0.007 |
| neg-M283T282 | Phenylpropanoids and polyketides | Wogonin | C10197 | 3.132 | 42.689 | 0.011 |
| pos-M177T197 | Phenylpropanoids and polyketides | 4-Methylumbelliferone | C03081 | 1.773 | 8.474 | 0.019 |
| neg-M253T239 | Phenylpropanoids and polyketides | Daidzein | C10208 | 2.251 | 13.524 | 0.022 |
| pos-M285T241 | Phenylpropanoids and polyketides | Biochanin A | C00814 | 1.898 | 13.695 | 0.024 |
| neg-M285T279 | Phenylpropanoids and polyketides | Isosakuranetin | C05334 | 1.432 | 6.323 | 0.024 |
| pos-M301T255 | Phenylpropanoids and polyketides | Chrysoeriol | C04293 | 2.213 | 11.030 | 0.035 |
| neg-M179T206 | Phenylpropanoids and polyketides | trans-Caffeic acid | C01197 | 2.408 | 0.086 | 0.044 |
| pos-M287T240 | Phenylpropanoids and polyketides | Luteolin | C01514 | 1.641 | 5.098 | 0.044 |
| neg-M301T255_1 | Phenylpropanoids and polyketides | Hesperetin | C01709 | 1.346 | 8.362 | 0.044 |
| pos-M372T223 | Phenylpropanoids and polyketides | Tamoxifen | C07108 | 1.888 | 9.214 | 0.044 |
| pos-M287T216 | Phenylpropanoids and polyketides | Datiscetin | C10036 | 1.645 | 4.956 | 0.049 |
| neg-M139T75 | Benzenoids | o-Fluorobenzoic acid | C02359 | 3.399 | 182.783 | 0.004 |
| neg-M123T201 | Benzenoids | 3-Hydroxybenzyl alcohol | C03351 | 2.780 | 0.174 | 0.004 |
| neg-M163T200 | Benzenoids | (3-Methylphenyl)methyl acetate | C07216 | 2.034 | 0.171 | 0.010 |
| neg-M119T204 | Benzenoids | 2-Methylbenzaldehyde | C07214 | 1.653 | 11.519 | 0.012 |
| pos-M138T90 | Benzenoids | Tyramine | C00483 | 2.867 | 16.937 | 0.014 |
| neg-M167T227_1 | Benzenoids | Vanillic acid | C06672 | 2.306 | 0.144 | 0.014 |
| pos-M325T196_2 | Benzenoids | Citalopram | C07572 | 2.274 | 0.155 | 0.014 |
| neg-M135T225 | Benzenoids | M-toluic Acid | C07211 | 2.039 | 0.233 | 0.019 |
| neg-M166T54 | Benzenoids | o-Nitrobenzoic acid | C16234 | 1.892 | 8.019 | 0.022 |
| pos-M121T87 | Benzenoids | Styrene Oxide | C02083 | 1.886 | 11.274 | 0.040 |
| neg-M127T141 | Benzenoids | 4-Chlorophenol | C02124 | 1.988 | 0.408 | 0.049 |
| neg-M195T48_2 | Organic oxygen compounds | D-Gluconic acid | C00257 | 1.840 | 5.481 | 0.015 |
| neg-M261T40 | Organic oxygen compounds | D-Mannitol 1-phosphate | C00644 | 1.634 | 3.871 | 0.017 |
| neg-M207T202 | Organic oxygen compounds | L-Kynurenine | C00328 | 1.563 | 0.309 | 0.028 |
| neg-M549T53 | Organic oxygen compounds | Maltotriose | C01835 | 2.769 | 25.033 | 0.032 |
| neg-M206T207 | Organic oxygen compounds | o-Aminobenzoylbrenztraubensaure | C01252 | 1.590 | 0.283 | 0.049 |
| pos-M205T47_2 | Organic nitrogen compounds | Ethambutol | C06984 | 3.745 | 346.202 | 0.001 |
| pos-M89T38_5 | Organic nitrogen compounds | Putrescine | C00134 | 2.038 | 5.364 | 0.011 |
| neg-M225T192_2 | Organic nitrogen compounds | Porphobilinogen | C00931 | 2.021 | 3.039 | 0.032 |
| pos-M104T39 | Organic nitrogen compounds | Choline | C00114 | 2.239 | 7.123 | 0.035 |
| neg-M97T279 | Organohalogen compounds | 1,2-Dichloroethane | C06752 | 1.409 | 2.948 | 0.015 |
| pos-M252T98 | Nucleosides, nucleotides, and analogues | Deoxyadenosine | C00559 | 2.162 | 10.897 | 0.028 |
| neg-M243T73 | Nucleosides, nucleotides, and analogues | Uridine | C00299 | 1.717 | 0.169 | 0.049 |
| neg-M189T158 | Organooxygen compounds | 3-Dehydroquinic acid | C00944 | 1.818 | 0.298 | 0.040 |
| neg-M179T52 | Carbohydrates | Allose | C01487 | 1.788 | 3.698 | 0.008 |
| **PTB_DM VS PTB.significant** | | | | | | |
| **ID** | **MS2superclass** | **MS2Metabolite** | **MS2kegg** | **VIP** | **FC** | **wilcox.test_**  **p.value_BHcorrect** |
| neg-M204T86 | Lipids and lipid-like molecules | 5-Acetamidovalerate | C03087 | 2.627 | 0.229 | 0.001 |
| pos-M118T91 | Lipids and lipid-like molecules | L-Norvaline | C01826 | 2.082 | 0.398 | 0.001 |
| neg-M263T266 | Lipids and lipid-like molecules | (+)-Abscisic acid | C06082 | 1.922 | 0.385 | 0.002 |
| pos-M246T205_2 | Lipids and lipid-like molecules | Acylcarnitine 5:0 | C02301 | 2.231 | 0.278 | 0.003 |
| neg-M487T286 | Lipids and lipid-like molecules | Ananasic acid | C12599 | 2.404 | 0.202 | 0.003 |
| neg-M528T272 | Lipids and lipid-like molecules | Glycochenodeoxycholic acid 7-sulfate | C15559 | 3.779 | 162.309 | 0.005 |
| neg-M129T79 | Lipids and lipid-like molecules | Citraconic acid | C02226 | 2.326 | 3.545 | 0.006 |
| pos-M315T261 | Lipids and lipid-like molecules | 12,13-Dihydroxy-9Z-octadecenoic acid | C14829 | 2.653 | 5.415 | 0.010 |
| pos-M132T52 | Lipids and lipid-like molecules | 6-Aminocaproic acid | C02378 | 2.908 | 0.170 | 0.011 |
| pos-M218T112 | Lipids and lipid-like molecules | L-Propionylcarnitine | C03017 | 2.352 | 0.242 | 0.022 |
| neg-M315T344 | Lipids and lipid-like molecules | 9,10-dihydroxyoctadecanoate | C15988 | 2.411 | 70.378 | 0.033 |
| neg-M227T277 | Lipids and lipid-like molecules | trans-Traumatic acid | C16308 | 2.482 | 0.106 | 0.033 |
| neg-M183T255 | Lipids and lipid-like molecules | Undecylenic acid | C13910 | 2.121 | 0.348 | 0.038 |
| pos-M269T79 | Organic acids and derivatives | His-Leu | C05010 | 2.330 | 0.267 | 0.001 |
| neg-M187T190 | Organic acids and derivatives | Gly-Leu | C02155 | 2.181 | 0.320 | 0.001 |
| pos-M220T190_2 | Organic acids and derivatives | Pantothenic acid | C00864 | 2.328 | 0.272 | 0.003 |
| pos-M197T192_1 | Organic acids and derivatives | L-Homophenylalanine | C17235 | 1.932 | 0.383 | 0.003 |
| neg-M88T193 | Organic acids and derivatives | 2-Aminopropanoic acid | C01401 | 1.950 | 0.431 | 0.006 |
| neg-M130T191 | Organic acids and derivatives | Beta-Leucine | C02486 | 1.822 | 0.407 | 0.007 |
| pos-M172T51 | Organic acids and derivatives | Tetrahydrodipicolinate | C03972 | 1.949 | 0.296 | 0.008 |
| pos-M130T78 | Organic acids and derivatives | L-5-Oxoproline | C01879 | 2.480 | 3.682 | 0.010 |
| pos-M198T105 | Organic acids and derivatives | N-Acetylhistidine | C02997 | 2.409 | 0.187 | 0.011 |
| neg-M116T96 | Organic acids and derivatives | 5-Aminopentanoic acid | C00431 | 1.907 | 0.357 | 0.013 |
| pos-M277T51 | Organic acids and derivatives | L-Saccharopine | C00449 | 2.328 | 0.361 | 0.013 |
| neg-M188T104 | Organic acids and derivatives | N-Acetylglutamic acid | C00624 | 1.901 | 0.219 | 0.015 |
| neg-M161T45 | Organic acids and derivatives | Diethyl dicarbonate | C11592 | 1.700 | 0.440 | 0.020 |
| pos-M176T48 | Organic acids and derivatives | L-Citrulline | C00327 | 1.760 | 0.478 | 0.026 |
| pos-M150T73_2 | Organic acids and derivatives | Methionine | C00073 | 1.555 | 0.434 | 0.029 |
| pos-M148T48 | Organic acids and derivatives | Glutamic acid | C00025 | 1.745 | 0.327 | 0.038 |
| pos-M116T195_2 | Organic acids and derivatives | Proline | C00148 | 1.662 | 0.465 | 0.038 |
| neg-M177T83 | Organic acids and derivatives | 2-Keto-3-deoxy-D-gluconic acid | C01216 | 1.468 | 0.495 | 0.038 |
| neg-M172T48 | Organic acids and derivatives | 2-acetamido-5-oxopentanoic acid | C01250 | 1.421 | 2.662 | 0.043 |
| pos-M132T104_1 | Organic acids and derivatives | Isoleucine | C00407 | 1.733 | 0.382 | 0.049 |
| neg-M182T201 | Organoheterocyclic compounds | Saccharin | C12283 | 1.477 | 2.645 | 0.008 |
| pos-M204T46 | Organoheterocyclic compounds | 3-Indolebutyric acid | C11284 | 2.257 | 0.277 | 0.010 |
| pos-M153T114 | Organoheterocyclic compounds | Xanthine | C00385 | 2.776 | 0.168 | 0.015 |
| pos-M135T204 | Organoheterocyclic compounds | 1-Isobenzofuranone | C09921 | 1.667 | 5.426 | 0.015 |
| pos-M137T93 | Organoheterocyclic compounds | Hypoxanthine | C00262 | 2.180 | 0.286 | 0.020 |
| pos-M86T104_2 | Organoheterocyclic compounds | Piperidine | C01746 | 1.788 | 0.361 | 0.020 |
| pos-M132T191_2 | Organoheterocyclic compounds | 3-Methylindole | C08313 | 1.623 | 0.471 | 0.038 |
| neg-M218T192 | Benzenoids | N-Phenyl-2-naphthylamine | C14694 | 1.874 | 0.433 | 0.011 |
| neg-M153T213 | Benzenoids | Gentisic acid | C00628 | 2.194 | 0.359 | 0.017 |
| neg-M196T210 | Benzenoids | p-Aminobenzoic acid | C00568 | 1.789 | 0.426 | 0.033 |
| neg-M123T201 | Benzenoids | 3-Hydroxybenzyl alcohol | C03351 | 2.899 | 0.148 | 0.033 |
| neg-M194T227 | Benzenoids | 2-Hydroxyhippuric acid | C07588 | 1.848 | 0.285 | 0.038 |
| neg-M171T188 | Benzenoids | 1-Naphthoic acid | C14091 | 1.722 | 0.353 | 0.043 |
| neg-M282T101 | Nucleosides, nucleotides, and analogues | Guanosine | C00387 | 3.278 | 0.149 | 0.006 |
| neg-M283T101 | Nucleosides, nucleotides, and analogues | Xanthosine | C01762 | 2.559 | 0.202 | 0.010 |
| neg-M243T84_1 | Nucleosides, nucleotides, and analogues | Uridine | C00299 | 2.942 | 0.243 | 0.011 |
| neg-M267T103 | Nucleosides, nucleotides, and analogues | Inosine | C00294 | 3.206 | 0.188 | 0.013 |
| pos-M282T77 | Nucleosides, nucleotides, and analogues | 1-Methyladenosine | C02494 | 2.156 | 0.221 | 0.043 |
| neg-M223T224 | Phenylpropanoids and polyketides | Sinapic acid | C00482 | 2.463 | 22.746 | 0.043 |
| neg-M345T247 | Phenylpropanoids and polyketides | Syringetin | C11620 | 1.844 | 0.160 | 0.043 |
| neg-M301T237 | Lignans, neolignans and related compounds | Enterodiol | C18166 | 2.012 | 0.233 | 0.029 |
