# Supplementary material for: Disorders of gut microbiota and fecal–serum metabolic patterns are associated with pulmonary tuberculosis and pulmonary tuberculosis comorbid type 2 diabetes mellitus
Source: Microbiol Spectr. 2025 Mar 14;13(8):e01772-24. doi: 10.1128/spectrum.01772-24 (PMC12323600; doi:10.1128/spectrum.01772-24)
Supplement: Table S3 — Pathway enrichment of fecal differential metabolites. [file spectrum.01772-24-s0008.docx]

**Table S3 Pathway enrichment of fecal differential metabolites**

| **PTB VS Health.idms2.pathway** | | | | |
| --- | --- | --- | --- | --- |
| **Pathway ID** | **Pathway** | **Rich factor** | **Pvalue** | **FDR** |
| map00400 | Phenylalanine, tyrosine and tryptophan biosynthesis | 0.171 | 0.00001 | 0.00018 |
| map00330 | Arginine and proline metabolism | 0.092 | 0.00034 | 0.00218 |
| map00250 | Alanine, aspartate and glutamate metabolism | 0.143 | 0.00040 | 0.00241 |
| map00380 | Tryptophan metabolism | 0.086 | 0.00052 | 0.00296 |
| map00340 | Histidine metabolism | 0.106 | 0.00068 | 0.00365 |
| map00232 | Caffeine metabolism | 0.190 | 0.00009 | 0.00085 |
| map01061 | Biosynthesis of phenylpropanoids | 0.097 | 0.00002 | 0.00028 |
| map01065 | Biosynthesis of alkaloids derived from histidine and purine | 0.114 | 0.00115 | 0.00553 |
| map01100 | Metabolic pathways | 0.040 | 0.00000 | 0.00000 |
| map01230 | Biosynthesis of amino acids | 0.102 | 0.00000 | 0.00002 |
| map01110 | Biosynthesis of secondary metabolites | 0.036 | 0.00096 | 0.00485 |
| map00564 | Glycerophospholipid metabolism | 0.115 | 0.00017 | 0.00130 |
| map02010 | ABC transporters | 0.111 | 0.00000 | 0.00000 |
| map00460 | Cyanoamino acid metabolism | 0.133 | 0.00007 | 0.00067 |
| map00430 | Taurine and hypotaurine metabolism | 0.091 | 0.01363 | 0.04111 |
| map00230 | Purine metabolism | 0.098 | 0.00004 | 0.00051 |
| map00970 | Aminoacyl-tRNA biosynthesis | 0.212 | 0.00000 | 0.00000 |
| map00260 | Glycine, serine and threonine metabolism | 0.120 | 0.00013 | 0.00110 |
| map00360 | Phenylalanine metabolism | 0.097 | 0.00023 | 0.00161 |
| map00290 | Valine, leucine and isoleucine biosynthesis | 0.130 | 0.00174 | 0.00792 |
| map00270 | Cysteine and methionine metabolism | 0.065 | 0.01400 | 0.04111 |
| map00220 | Arginine biosynthesis | 0.087 | 0.01542 | 0.04385 |
| map00261 | Monobactam biosynthesis | 0.103 | 0.00190 | 0.00823 |
| map02030 | Bacterial chemotaxis | 0.167 | 0.00755 | 0.02454 |
| map01064 | Biosynthesis of alkaloids derived from ornithine, lysine and nicotinic acid | 0.075 | 0.00435 | 0.01647 |
| map01063 | Biosynthesis of alkaloids derived from shikimate pathway | 0.056 | 0.00568 | 0.02069 |
| map01502 | Vancomycin resistance | 0.083 | 0.01733 | 0.04778 |
| map01210 | 2-Oxocarboxylic acid metabolism | 0.060 | 0.00370 | 0.01464 |
| map00591 | Linoleic acid metabolism | 0.107 | 0.00368 | 0.01464 |
| map00072 | Synthesis and degradation of ketone bodies | 0.167 | 0.00755 | 0.02454 |
| map00760 | Nicotinate and nicotinamide metabolism | 0.073 | 0.00856 | 0.02685 |
| map00410 | beta-Alanine metabolism | 0.219 | 0.00000 | 0.00001 |
| map00473 | D-Alanine metabolism | 0.167 | 0.00755 | 0.02454 |
| **PTB_DM VS Health.idms2.pathway** | | | | |
| **Pathway ID** | **Pathway** | **Rich factor** | **Pvalue** | **FDR** |
| map00400 | Phenylalanine, tyrosine and tryptophan biosynthesis | 0.171 | 0.00007 | 0.00109 |
| map00340 | Histidine metabolism | 0.128 | 0.00046 | 0.00450 |
| map00250 | Alanine, aspartate and glutamate metabolism | 0.143 | 0.00134 | 0.01008 |
| map00380 | Tryptophan metabolism | 0.086 | 0.00292 | 0.01488 |
| map00330 | Arginine and proline metabolism | 0.079 | 0.00775 | 0.03581 |
| map00232 | Caffeine metabolism | 0.190 | 0.00033 | 0.00447 |
| map01061 | Biosynthesis of phenylpropanoids | 0.136 | 0.00000 | 0.00001 |
| map01065 | Biosynthesis of alkaloids derived from histidine and purine | 0.171 | 0.00007 | 0.00109 |
| map01100 | Metabolic pathways | 0.049 | 0.00000 | 0.00000 |
| map01230 | Biosynthesis of amino acids | 0.134 | 0.00000 | 0.00000 |
| map01110 | Biosynthesis of secondary metabolites | 0.044 | 0.00135 | 0.01008 |
| map00564 | Glycerophospholipid metabolism | 0.115 | 0.00087 | 0.00766 |
| map02010 | ABC transporters | 0.087 | 0.00038 | 0.00447 |
| map00430 | Taurine and hypotaurine metabolism | 0.182 | 0.00041 | 0.00447 |
| map00460 | Cyanoamino acid metabolism | 0.111 | 0.00215 | 0.01303 |
| map00230 | Purine metabolism | 0.087 | 0.00172 | 0.01113 |
| map00970 | Aminoacyl-tRNA biosynthesis | 0.154 | 0.00002 | 0.00050 |
| map00310 | Lysine degradation | 0.096 | 0.00451 | 0.02189 |
| map00350 | Tyrosine metabolism | 0.077 | 0.00891 | 0.03929 |
| map00650 | Butanoate metabolism | 0.119 | 0.00149 | 0.01033 |
| map00660 | C5-Branched dibasic acid metabolism | 0.125 | 0.00248 | 0.01417 |
| map00365 | Furfural degradation | 0.200 | 0.00280 | 0.01488 |
| **PTB_DM VS PTB.idms2.pathway** | | | | |
| **Pathway ID** | **Pathway** | **Rich factor** | **Pvalue** | **FDR** |
| map00400 | Phenylalanine, tyrosine and tryptophan biosynthesis | 0.086 | 0.00508 | 0.03047 |
| map00232 | Caffeine metabolism | 0.190 | 0.00005 | 0.00118 |
| map01065 | Biosynthesis of alkaloids derived from histidine and purine | 0.086 | 0.00508 | 0.03047 |
| map01100 | Metabolic pathways | 0.033 | 0.00000 | 0.00017 |
| map00564 | Glycerophospholipid metabolism | 0.096 | 0.00056 | 0.00472 |
| map00460 | Cyanoamino acid metabolism | 0.089 | 0.00196 | 0.01372 |
| map00230 | Purine metabolism | 0.087 | 0.00008 | 0.00118 |
| map00970 | Aminoacyl-tRNA biosynthesis | 0.115 | 0.00007 | 0.00118 |
| map00220 | Arginine biosynthesis | 0.174 | 0.00008 | 0.00118 |
| map00310 | Lysine degradation | 0.096 | 0.00056 | 0.00472 |
| map00290 | Valine, leucine and isoleucine biosynthesis | 0.130 | 0.00103 | 0.00789 |
| map01230 | Biosynthesis of amino acids | 0.102 | 0.00000 | 0.00001 |
| map01210 | 2-Oxocarboxylic acid metabolism | 0.067 | 0.00033 | 0.00345 |
| map00591 | Linoleic acid metabolism | 0.143 | 0.00021 | 0.00248 |
| map00473 | D-Alanine metabolism | 0.167 | 0.00575 | 0.03219 |
| map00480 | Glutathione metabolism | 0.079 | 0.00684 | 0.03589 |
