# Supplementary material for: Disorders of gut microbiota and fecal–serum metabolic patterns are associated with pulmonary tuberculosis and pulmonary tuberculosis comorbid type 2 diabetes mellitus
Source: Microbiol Spectr. 2025 Mar 14;13(8):e01772-24. doi: 10.1128/spectrum.01772-24 (PMC12323600; doi:10.1128/spectrum.01772-24)
Supplement: Table S4 — Differential metabolites of serum metabolome. [file spectrum.01772-24-s0009.docx]

**Table S4 Differential metabolites of serum metabolome**

| **PTB VS Health.significant** | | | | | | |
| --- | --- | --- | --- | --- | --- | --- |
| **ID** | **MS2superclass** | **MS2Metabolite** | **MS2kegg** | **VIP** | **FC** | **wilcox.test_**  **p.value_BHcorrect** |
| neg-M151T64 | Organoheterocyclic compounds | Xanthine | C00385 | 2.427 | 0.407 | 0.040 |
| pos-M148T49 | Organic acids and derivatives | Glutamic acid | C00025 | 2.603 | 2.680 | 0.076 |
| neg-M512T200 | Lipids and lipid-like molecules | LysoPC 14:0 | C04230 | 2.208 | 0.433 | 0.095 |
| pos-M76T50 | Organic nitrogen compounds | Trimethylamine N-oxide | C01104 | 1.802 | 0.359 | 0.116 |
| neg-M166T354 | Organoheterocyclic compounds | 2(3H)-Benzothiazolethione | C14437 | 1.701 | 0.481 | 0.125 |
| neg-M301T317 | Lipids and lipid-like molecules | Eicosapentaenoic acid | C06428 | 1.679 | 0.498 | 0.141 |
| pos-M401T458 | Lipids and lipid-like molecules | 7.alpha.-Hydroxy-4-cholesten-3-one | C05455 | 1.815 | 2.084 | 0.141 |
| neg-M179T134 | Organoheterocyclic compounds | Paraxanthine | C13747 | 2.441 | 0.258 | 0.188 |
| pos-M615T426 | Lipids and lipid-like molecules | SM 28:3; SM(d14:2/14:1) | C00550 | 1.881 | 0.487 | 0.319 |
| pos-M195T139 | Organoheterocyclic compounds | Caffeine | C07481 | 2.331 | 0.098 | 0.347 |
| **PTB_DM VS Health.significant** | | | | | | |
| **ID** | **MS2superclass** | **MS2Metabolite** | **MS2kegg** | **VIP** | **FC** | **wilcox.test_**  **p.value_BHcorrect** |
| neg-M179T50 | Unknown | Allose | C01487 | 1.911 | 2.254 | 0.075 |
| neg-M391T198 | Lipids and lipid-like molecules | Deoxycholic acid | C04483 | 3.209 | 0.288 | 0.083 |
| pos-M124T119 | Organoheterocyclic compounds | Pyrazinamide | C01956 | 3.615 | 43.191 | 0.092 |
| neg-M103T119 | Organic oxygen compounds | Acetone | C00207 | 2.102 | 2.442 | 0.100 |
| pos-M187T53 | Unknown | 4-Isoxazolepropanoic acid, .alpha.-amino-2,3-dihydro-5-methyl-3-oxo- | C13672 | 2.511 | 0.454 | 0.100 |
| pos-M286T201 | Alkaloids and derivatives | Piperine | C03882 | 3.108 | 0.083 | 0.107 |
| neg-M179T134 | Organoheterocyclic compounds | Paraxanthine | C13747 | 2.615 | 0.264 | 0.160 |
| pos-M369T460 | Lipids and lipid-like molecules | Cholesterol | C00187 | 1.604 | 2.016 | 0.176 |
| neg-M178T142 | Benzenoids | Hippuric acid | C01586 | 2.808 | 0.258 | 0.176 |
| neg-M129T65_1 | Lipids and lipid-like molecules | Mesaconic acid | C01732 | 1.502 | 2.828 | 0.188 |
| pos-M76T50 | Organic nitrogen compounds | Trimethylamine N-oxide | C01104 | 2.321 | 0.352 | 0.215 |
| pos-M468T200 | Lipids and lipid-like molecules | LysoPC 14:0 | C04230 | 2.341 | 0.471 | 0.223 |
| pos-M180T143 | Phenylpropanoids and polyketides | Umbelliferone | C09315 | 2.295 | 0.281 | 0.223 |
| pos-M777T482 | Lipids and lipid-like molecules | PC(18:3(6Z,9Z,12Z)/18:4(6Z,9Z,12Z,15Z)) | C00157 | 1.361 | 2.090 | 0.288 |
| neg-M528T178 | Lipids and lipid-like molecules | N-[(3a,5b,7a)-3-hydroxy-24-oxo-7-(sulfooxy)cholan-24-yl]-Glycine | C15559 | 2.646 | 7.066 | 0.324 |
| neg-M103T101 | Organic acids and derivatives | (S)-3-Hydroxybutyric acid | C03197 | 2.203 | 4.303 | 0.324 |
| pos-M355T155 | Organoheterocyclic compounds | Flumioxazin | C11035 | 1.466 | 2.829 | 0.324 |
| pos-M593T433 | Lipids and lipid-like molecules | SM 26:0; SM(d14:0/12:0) | C00550 | 1.829 | 0.472 | 0.338 |
| neg-M191T62 | Organic acids and derivatives | Citric acid | C00158 | 1.664 | 7.630 | 0.353 |
| pos-M183T178 | Benzenoids | Benzophenone | C06354 | 1.640 | 3.574 | 0.421 |
| **PTB_DM VS PTB.significant** | | | | | | |
| **ID** | **MS2superclass** | **MS2Metabolite** | **MS2kegg** | **VIP** | **FC** | **wilcox.test_**  **p.value_BHcorrect** |
| pos-M187T53 | Unknown | 4-Isoxazolepropanoic acid, .alpha.-amino-2,3-dihydro-5-methyl-3-oxo- | C13672 | 3.924 | 0.373 | 0.635 |
| pos-M286T201 | Alkaloids and derivatives | Piperine | C03882 | 4.123 | 0.109 | 0.635 |
| pos-M124T119 | Organoheterocyclic compounds | Pyrazinamide | C01956 | 5.152 | 38.879 | 0.715 |
| pos-M176T157 | Organoheterocyclic compounds | 3-Indoleacetic acid | C00954 | 3.087 | 0.475 | 0.715 |
| neg-M103T119 | Organic oxygen compounds | Acetone | C00207 | 3.082 | 2.208 | 0.861 |
| pos-M169T170 | Organic oxygen compounds | 2,6-Dimethoxy-1,4-benzoquinone | C10331 | 3.563 | 0.442 | 0.945 |
| neg-M407T184 | Lipids and lipid-like molecules | Cholic acid | C00695 | 2.752 | 2.376 | 1.000 |
| neg-M762T355 | Lipids and lipid-like molecules | PE(18:2(9Z,12Z)/20:4(5Z,8Z,11Z,14Z)) | C00350 | 2.626 | 3.712 | 1.000 |
| neg-M183T150 | Benzenoids | 4-Hydroxybenzoic acid | C00156 | 2.601 | 0.453 | 1.000 |
