# Supplementary material for: Disorders of gut microbiota and fecal–serum metabolic patterns are associated with pulmonary tuberculosis and pulmonary tuberculosis comorbid type 2 diabetes mellitus
Source: Microbiol Spectr. 2025 Mar 14;13(8):e01772-24. doi: 10.1128/spectrum.01772-24 (PMC12323600; doi:10.1128/spectrum.01772-24)
Supplement: Table S5 — Pathway enrichment of serum differential metabolites. [file spectrum.01772-24-s0010.docx]

**Table S5 Pathway enrichment of serum differential metabolites**

| **PTB VS Health.idms2.pathway** | | | | |
| --- | --- | --- | --- | --- |
| **Pathway ID** | **Pathway** | **Rich factor** | **Pvalue** | **FDR** |
| map00600 | Sphingolipid metabolism | 0.040 | 0.00143 | 0.00274 |
| map00120 | Primary bile acid biosynthesis | 0.021 | 0.00501 | 0.00707 |
| map00564 | Glycerophospholipid metabolism | 0.019 | 0.00611 | 0.00814 |
| map00250 | Alanine, aspartate and glutamate metabolism | 0.036 | 0.00179 | 0.00331 |
| map00232 | Caffeine metabolism | 0.143 | 0.00000 | 0.00000 |
| map00660 | C5-Branched dibasic acid metabolism | 0.031 | 0.00234 | 0.00401 |
| map04071 | Sphingolipid signaling pathway | 0.063 | 0.00058 | 0.00146 |
| map05231 | Choline metabolism in cancer | 0.091 | 0.00027 | 0.00085 |
| map04742 | Taste transduction | 0.031 | 0.00234 | 0.00401 |
| map00220 | Arginine biosynthesis | 0.043 | 0.00121 | 0.00241 |
| map00340 | Histidine metabolism | 0.021 | 0.00501 | 0.00707 |
| map00330 | Arginine and proline metabolism | 0.013 | 0.01274 | 0.01569 |
| map00650 | Butanoate metabolism | 0.024 | 0.00401 | 0.00621 |
| map00630 | Glyoxylate and dicarboxylate metabolism | 0.016 | 0.00833 | 0.01053 |
| map00910 | Nitrogen metabolism | 0.053 | 0.00082 | 0.00179 |
| map01200 | Carbon metabolism | 0.009 | 0.02660 | 0.03040 |
| map01230 | Biosynthesis of amino acids | 0.008 | 0.03362 | 0.03667 |
| map01210 | 2-Oxocarboxylic acid metabolism | 0.007 | 0.03712 | 0.03791 |
| map01100 | Metabolic pathways | 0.004 | 0.04255 | 0.04255 |
| map01040 | Biosynthesis of unsaturated fatty acids | 0.019 | 0.00657 | 0.00853 |
| map00471 | D-Glutamine and D-glutamate metabolism | 0.083 | 0.00032 | 0.00090 |
| map00430 | Taurine and hypotaurine metabolism | 0.045 | 0.00110 | 0.00230 |
| map00480 | Glutathione metabolism | 0.026 | 0.00329 | 0.00527 |
| map00230 | Purine metabolism | 0.011 | 0.01836 | 0.02149 |
| map04974 | Protein digestion and absorption | 0.021 | 0.00501 | 0.00707 |
| map04964 | Proximal tubule bicarbonate reclamation | 0.059 | 0.00065 | 0.00157 |
| map04720 | Long-term potentiation | 0.143 | 0.00010 | 0.00082 |
| map04724 | Glutamatergic synapse | 0.125 | 0.00014 | 0.00084 |
| map04727 | GABAergic synapse | 0.111 | 0.00017 | 0.00084 |
| map04730 | Long-term depression | 0.111 | 0.00017 | 0.00084 |
| map02010 | ABC transporters | 0.008 | 0.03313 | 0.03667 |
| map04068 | FoxO signaling pathway | 0.200 | 0.00005 | 0.00078 |
| map04072 | Phospholipase D signaling pathway | 0.077 | 0.00038 | 0.00100 |
| map00970 | Aminoacyl-tRNA biosynthesis | 0.019 | 0.00611 | 0.00814 |
| map05230 | Central carbon metabolism in cancer | 0.027 | 0.00312 | 0.00517 |
| map00860 | Porphyrin and chlorophyll metabolism | 0.007 | 0.03712 | 0.03791 |
| map00524 | Neomycin, kanamycin and gentamicin biosynthesis | 0.012 | 0.01440 | 0.01728 |
| map05016 | Huntington's disease | 0.333 | 0.00001 | 0.00035 |
| map05014 | Amyotrophic lateral sclerosis (ALS) | 0.100 | 0.00022 | 0.00085 |
| map05030 | Cocaine addiction | 0.143 | 0.00010 | 0.00082 |
| map05033 | Nicotine addiction | 0.143 | 0.00010 | 0.00082 |
| map05031 | Amphetamine addiction | 0.111 | 0.00017 | 0.00084 |
| map05034 | Alcoholism | 0.100 | 0.00022 | 0.00085 |
| map04713 | Circadian entrainment | 0.091 | 0.00027 | 0.00085 |
| map04721 | Synaptic vesicle cycle | 0.083 | 0.00032 | 0.00090 |
| map04723 | Retrograde endocannabinoid signaling | 0.053 | 0.00082 | 0.00179 |
| map04540 | Gap junction | 0.091 | 0.00027 | 0.00085 |
| map04080 | Neuroactive ligand-receptor interaction | 0.008 | 0.03510 | 0.03744 |
| **PTB_DM VS Health.idms2.pathway** | | | | |
| **Pathway ID** | **Pathway** | **Rich factor** | **Pvalue** | **FDR** |
| map04071 | Sphingolipid signaling pathway | 0.063 | 0.00144 | 0.00742 |
| map05231 | Choline metabolism in cancer | 0.182 | 0.00001 | 0.00011 |
| map00250 | Alanine, aspartate and glutamate metabolism | 0.036 | 0.00443 | 0.00886 |
| map00232 | Caffeine metabolism | 0.048 | 0.00250 | 0.00886 |
| map00660 | C5-Branched dibasic acid metabolism | 0.031 | 0.00576 | 0.00988 |
| map00600 | Sphingolipid metabolism | 0.040 | 0.00354 | 0.00886 |
| map00120 | Primary bile acid biosynthesis | 0.021 | 0.01218 | 0.01686 |
| map00564 | Glycerophospholipid metabolism | 0.038 | 0.00077 | 0.00679 |
| map04742 | Taste transduction | 0.031 | 0.00576 | 0.00988 |
| map05200 | Pathways in cancer | 0.037 | 0.00412 | 0.00886 |
| map05230 | Central carbon metabolism in cancer | 0.027 | 0.00766 | 0.01199 |
| map00360 | Phenylalanine metabolism | 0.014 | 0.02733 | 0.03280 |
| map00020 | Citrate cycle (TCA cycle) | 0.050 | 0.00226 | 0.00886 |
| map00630 | Glyoxylate and dicarboxylate metabolism | 0.033 | 0.00123 | 0.00738 |
| map00640 | Propanoate metabolism | 0.021 | 0.01268 | 0.01690 |
| map00051 | Fructose and mannose metabolism | 0.019 | 0.01588 | 0.01972 |
| map01200 | Carbon metabolism | 0.018 | 0.00697 | 0.01141 |
| map00072 | Synthesis and degradation of ketone bodies | 0.167 | 0.00018 | 0.00221 |
| map00591 | Linoleic acid metabolism | 0.036 | 0.00443 | 0.00886 |
| map00100 | Steroid biosynthesis | 0.020 | 0.01371 | 0.01763 |
| map00590 | Arachidonic acid metabolism | 0.013 | 0.02949 | 0.03424 |
| map04975 | Fat digestion and absorption | 0.077 | 0.00094 | 0.00679 |
| map04977 | Vitamin digestion and absorption | 0.026 | 0.00849 | 0.01273 |
| map04976 | Bile secretion | 0.020 | 0.00480 | 0.00909 |
| map04925 | Aldosterone synthesis and secretion | 0.042 | 0.00326 | 0.00886 |
| map04913 | Ovarian steroidogenesis | 0.042 | 0.00326 | 0.00886 |
| map04922 | Glucagon signaling pathway | 0.038 | 0.00382 | 0.00886 |
| map04723 | Retrograde endocannabinoid signaling | 0.053 | 0.00204 | 0.00886 |
| map00981 | Insect hormone biosynthesis | 0.040 | 0.00354 | 0.00886 |
| map00592 | alpha-Linolenic acid metabolism | 0.024 | 0.00980 | 0.01411 |
| map05217 | Basal cell carcinoma | 1.000 | 0.00000 | 0.00000 |
| **PTB_DM VS PTB.idms2.pathway** | | | | |
| **Pathway ID** | **Pathway** | **Rich factor** | **Pvalue** | **FDR** |
| map00120 | Primary bile acid biosynthesis | 0.021 | 0.00214 | 0.00390 |
| map00564 | Glycerophospholipid metabolism | 0.019 | 0.00261 | 0.00406 |
| map00360 | Phenylalanine metabolism | 0.014 | 0.00496 | 0.00695 |
| map00380 | Tryptophan metabolism | 0.012 | 0.00625 | 0.00796 |
| map00640 | Propanoate metabolism | 0.021 | 0.00223 | 0.00390 |
| map01100 | Metabolic pathways | 0.003 | 0.00705 | 0.00822 |
| map00563 | Glycosylphosphatidylinositol (GPI)-anchor biosynthesis | 0.333 | 0.00001 | 0.00003 |
| map00072 | Synthesis and degradation of ketone bodies | 0.167 | 0.00003 | 0.00009 |
| map00130 | Ubiquinone and other terpenoid-quinone biosynthesis | 0.011 | 0.00768 | 0.00827 |
| map04976 | Bile secretion | 0.010 | 0.00906 | 0.00906 |
| map04723 | Retrograde endocannabinoid signaling | 0.053 | 0.00034 | 0.00080 |
| map04140 | Autophagy - animal | 0.250 | 0.00001 | 0.00004 |
| map05130 | Pathogenic Escherichia coli infection | 1.000 | 0.00000 | 0.00000 |
| map07110 | Benzoic acid family | 0.500 | 0.00000 | 0.00001 |
